# Cooperative stabilization mechanism and electrostatic evolution in the zero dimensional arsenolite cluster aggregates: a first principles investigation

**DOI:** 10.1039/d6ra08223h

**Published:** 2026-10-09

**Authors:** Basma Ali, Abdul Majid, Muhammad Tayyab Raza, Ali Hamza, Waheed Almasry, Muhammad Usman, Sajjad Haider

**Affiliations:** a Department of Physics, University of Gujrat Gujrat 50700 Pakistan abdulmajid40@yahoo.com; b Department of Physics, Gachon University 1342 Seongnam-daero, Sujeong-gu Seongnam-si Gyeonggi-do South Korea ali1214@gachon.ac.kr; c Department of Chemical Engineering, King Saud University P.O.Box 800 Riyadh 11421 Saudi Arabia; d Research School of Chemistry, Australian National University ACT 2601 Australia

## Abstract

Aggregation mechanism of molecular cage compounds is important in the understanding of cooperative intermolecular interactions which dictate the formation and stability of molecular solids. In this work, a comprehensive first-principles investigations are performed to explore the structural evolution, energetic stability, electronic properties, electrostatic interactions and trajectory based dynamical analysis of the arsenolite (As_4_O_6_) cluster series (C_1_–C_5_). The structural relaxation and electronic structure calculations are performed using density functional theory (DFT). The thermal stability of the clusters is studied by molecular dynamics (MD) simulations with finite temperature trajectory analysis. The structural descriptors, energetic analysis, reactivity descriptors, charge analyses and molecular electrostatic potential (ΔMEP) analyses are systematically employed to elucidate the aggregation mechanism. These results show that the evolution of clusters is thermodynamically favorable and this evolution is progressively associated with a cooperative stabilization without altering their intrinsic cage geometry. The entire optimized aggregates are also shown to be thermal robust using MD simulations assisted by python trajectories. In this study, the cooperative stabilization mechanism for the evolution of As_4_O_6_ clusters is established and fundamental theoretical insights into the self-assembly of molecular cage systems are provided which can form the rational base for the design of cage based molecular crystals and supramolecular materials.

## Abstract 2

First-principles calculations trace how intact Cn = (As_4_O_6_)*_n_*, *n* = 1–5 cages cooperatively assemble, revealing progressive charge redistribution and evolving inter-cage electrostatic network while retaining thermal stability.

## Graphical abstract

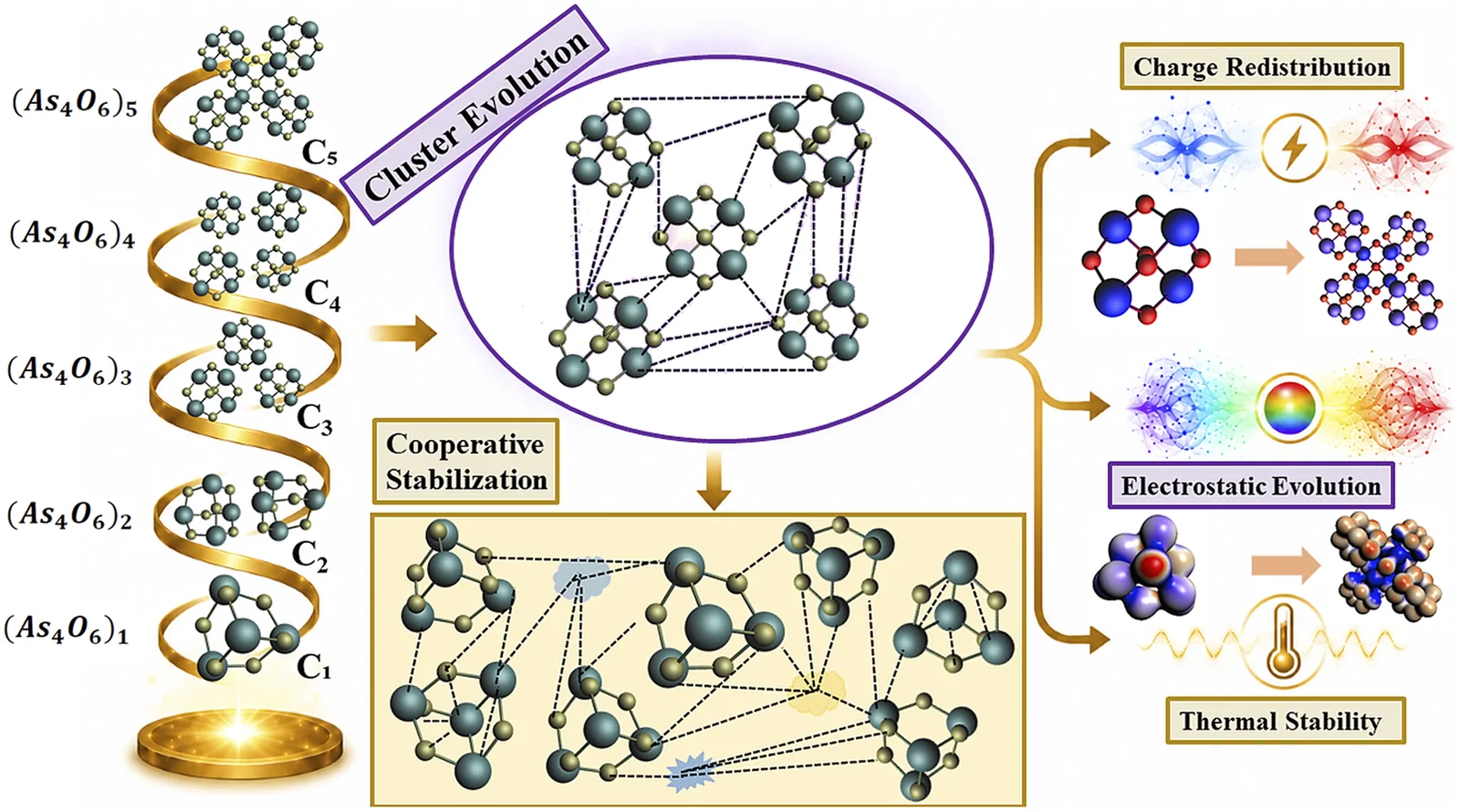

## 1. Introduction

In recent years zero-dimensional (0D) molecular systems have been occupying a special place in the field of inorganic chemistry since they are the basic building blocks from which more complex molecular aggregates and crystal architectures are formed.^1–3^ The term ‘0D’ is used here to denote the finite, isolated cluster regime (C_1_–C_5_) that is studied in contrast to the periodic 0D crystal systems (metal-halide perovskites) where discrete 0D units are embedded in an extended lattice.^4^ The molecular identity is preserved in the individual As_4_O_6_ cages in 0D molecular systems. Weak inter-cage interactions however can lead to emergent structural and electronic effects on aggregation that are not present in the individual cages. This study's focus is on how molecular individuality and aggregation-driven behavior interact.^5^ These investigations play an essential role in understanding the molecular basis of stability, self-organization and structure–property relationships in inorganic materials.^6^ Among the various molecular materials, inorganic molecular crystals (IMCs) are attractive because of their specific structure and physicochemical properties.^7^ IMCs consist of molecular building blocks which remain molecular throughout an ordered crystalline structure.^8^ The 0D IMCs in the form of the thin films or the quantum dots are creating a diverse molecular materials including C_60_, metal-halide perovskites and metal chalcogenides.^9–11^ Unlike traditional inorganic solids, in which the structural integrity and physical properties are controlled by continuous covalent, ionic or metallic networks, the structural integrity and physical properties of IMCs are significantly affected by intermolecular interactions between individual molecular units.^12^ Such interactions are frequently responsible for crystal packing, thermodynamics stability, polymorphism and molecular recognition phenomena.^13^ Hence, it is crucial to understand the relationship between isolated and aggregated molecular species to achieve a full understanding of inorganic molecular materials.^14^ One of the most significant subclass of IMCs are cage-like molecular systems.^15^ The molecular cages possess well-defined 3-D geometries, high structural rigidity and interesting electronic environments, making them attractive for a wide range of applications.^16^ Host–guest chemistry, molecular recognition, gas sensing, catalysis, optoelectronic materials, crystal engineering and nanotechnology have been thoroughly explored for cage architectures.^17–19^

Molecular cages are not only technologically relevant, they also represent optimal model systems for the study of fundamental questions related to aggregation and self-assembly of molecules.^20^ The discrete and geometrically confined architecture of their structures allows for systematic studies of the evolution of intermolecular interactions with growing size of the assembly and the emergence of collective stabilization from individual molecular building blocks.^17,21^ As the size of the molecular assembly becomes larger, progressive changes in structural adaptation, energetic stabilization, charge redistribution and changes in electronic properties can occur.^22,23^ It is especially interesting to study these cooperative effects as they form the molecular basis of supramolecular organization, leading to ordered crystalline matter in the final step.^24^ However, size-dependent studies of molecular ensembles yield a meaningful opportunity to detect aggregation motifs, to assess the strength of stabilization forces and to characterize the continuum from molecular to condensed molecular systems.^25^ As_4_O_6_ is a 0D molecular solid with independent cage like molecules of 4 arsenic and 6 oxygen atoms. Individual cages are stabilized by strong covalent As–O bonds, whereas intermolecular interactions are weak, thereby making arsenolite an ideal model system for the study of the balance between the molecular stability and assembly effects.^26^ The molecular nature of arsenolite has led to extensive studies of its mineralogical, crystal chemistry, high-pressure physics and theoretical materials science properties, including extensive reports on its structural, vibrational, compressibility and electronic properties.^27^ Recently, As_4_O_6_ has been explored by the density functional theory (DFT) calculation as a promising inorganic molecular framework for the Li-ion and Mg-ion battery application. In this study, the material was treated as a crystalline host structure with Li and Mg ions intercalated into the periodic As_4_O_6_ lattice and its electrochemical properties, including ion storage capacity, diffusion kinetics and voltage characteristics, were studied. Importantly, this work mainly deal with the host–guest electrochemical interactions under periodic boundary conditions, in which the electronic properties were obtained from the extended crystal environment.^28^ The present study, however, examines the intrinsic behavior of finite (As_4_O_6_)_*n*_ cluster assemblies (C_1_–C_5_) in the absence of guest species and periodic constraints. This enables us to isolate and study the basic inter-cage electronic interactions, the charge redistribution and the cooperative stabilization effects which are not available in the periodic intercalation scheme.^29–31^ When the number of molecular cages in an assembly grows, some properties may deviate significantly from the properties of individual cages and new stabilization mechanisms and collective behavior emerge. These systems can thus be regarded as molecular-scale “bridges” linking individual building blocks with bulk crystalline structures.^32^ Systematic examination of assemblies of varying size allows the accumulation of intermolecular interactions, the evolution of structural organization and the cooperative effects that lead to the overall stability of the system to be revealed.^32^ Recent progress in DFT has enabled the study of such phenomena in an atomistic sense.^33^ The modern quantum-chemical techniques give detailed information about changes in geometry, stabilization of the molecule, changes in electronic structure, redistribution of charges and intermolecular interaction.^34^ In addition, molecular dynamics (MD) simulations allow for the study of finite-temperature thermal stability and persistence of molecular assemblies.^35^ In combination, these methods offer a strong foundation to examine the basic processes involved in the evolution of 0D molecular systems.^36–39^ A systematic investigation of cage structures, C_*n*_ = (As_4_O_6_)_*n*_, *n* = 1–5 structural units, is carried out by DFT and MD calculations. The focus of this work is to find out whether the size dependent evolution of the C_*n*_ is a genuine cooperative stabilization or just a coincidence of cluster size and to test this by performing a combined structural, energetic, electronic, electrostatic and dynamical analysis.

## 2. Computational details

First-principles based computational strategy was employed to obtain a comprehensive understanding of cooperative stabilization and the evolution of the structural, energetic, electronic and dynamic properties of the C_*n*_ = (As_4_O_6_)_*n*_, *n* = 1–5 series.^40,41^ The calculations were carried out with the Amsterdam Density Functional (ADF) engine implemented in the Amsterdam Modeling Suite (AMS) while MD simulations were performed with the Density Functional Tight Binding (DFTB) engine within the same computational framework.^42–50^ The combination of these two quantum mechanical techniques beneficial to calculate the thermally fluctuating properties of molecular aggregates of larger size.^51^ The geometry of the isolated As_4_O_6_ cage and the progressively aggregated clusters (C_2_–C_5_) were fully optimized using DFT at the B3LYP-D3(BJ)/TZP level of theory. B3LYP exchange-correlation functional was selected because of its well-established capability to accurately reproduce the structural and electronic properties.^52^ The Grimme D3 dispersion correction with Becke–Johnson damping [D3(BJ)] is crucial for accounting for the weaker van der Waals interactions that drive the formation of adjacent As_4_O_6_ cages.^53,54^ An accurate description of the dispersion force is essential in order to obtain reliable interaction energies due to molecular aggregation induced mainly by non-covalent interactions. A triple-ζ polarized (TZP) Slater-type orbital basis set was employed without applying the frozen-core approximation to ensure an accurate all-electron description.^55^ The optimized geometries were used to extract the structural descriptors such as standard deviation of bond lengths, distortion indices which were extracted to investigate the structural evolution during cluster evolution. The structural distortion index (DI) was determined as,^56^1
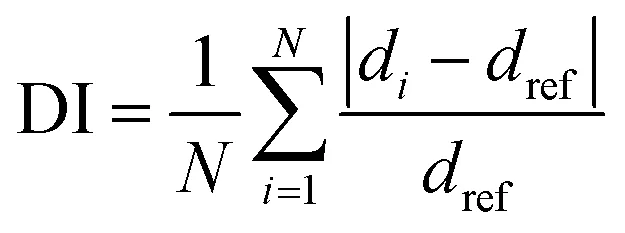
where *d*_*i*_ is the As–O bond length of the individual bond and *d*_ref_ is the As–O bond length of the optimized isolated As_4_O_6_ cage. The standard deviation (*σ*) can be calculated as,2
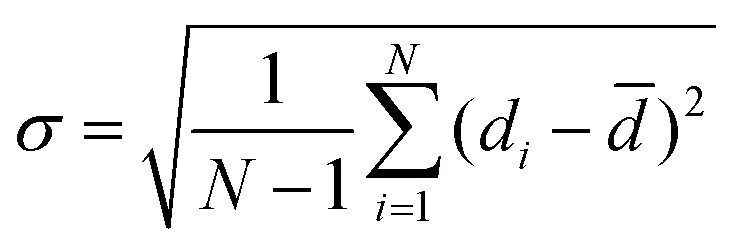


This is a quantitative measure of the deviation of each bond length from mean value and hence a valuable indicator of structural changes upon aggregation.^57^ The total bonding energies calculated from the optimized structures were then used to determine the thermodynamic driving force for cluster formation by computing a number of energetic descriptors such as binding energy per cage, relative energy and stabilization energy.^58^ These are the complementary energetic quantities related to the stability of each aggregate, the energetic contribution of each next cage and the evolution of the cooperative stabilization in the cluster series.^59,60^ The incremental energy was calculated as,3
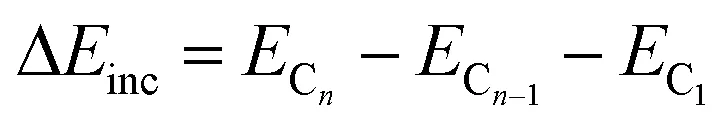
where *E*_C_*n*__ is the total electronic energy of the optimized n-cage cluster, as total bonding energy, *E*_C_*n*−1__ the total electronic energy of the (*n* − 1) cage cluster and *E*_C_1__ is the energy of the isolated As_4_O_6_ cage. The binding energy per cage was found to be,4
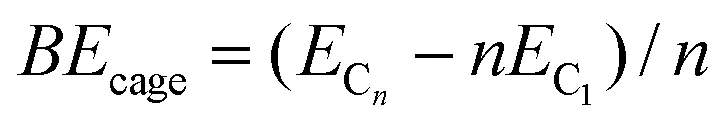


Single-point (SP) calculations are also performed to calculate the electronic structure, reliable frontier molecular orbital energies (FMO) and the molecular electron density which is necessary for further analyses.^61^ The energies of the highest occupied molecular orbital (HOMO) and the lowest unoccupied molecular orbital (LUMO) were extracted and the HOMO–LUMO (HL) gap was calculated as *E*_g_ = *E*_LUMO_ − *E*_HOMO_, which is an important descriptor of electronic stability, charge-transfer characteristics and chemical reactivity.^62^ To obtain a deeper understanding of the electronic response accompanying cluster aggregation, several descriptors were evaluated using Koopmans' approximation.^63^ The ionization potential (IP = −*E*_HOMO_) and electron affinity (EA = −*E*_LUMO_) were estimated from FMO energies. The electrophilicity index (*ω*), global hardness (*η*), chemical potential (*µ*) and global softness (*S*) are calculated using 
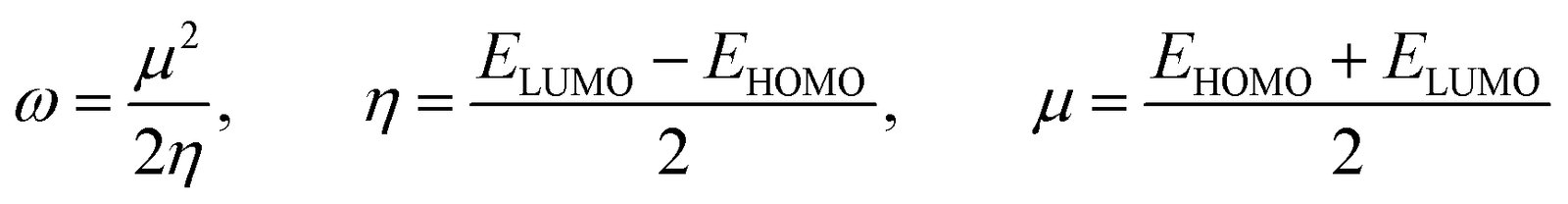
 and *S* = 1/2*η* (following original Parr–Pearson formulation) expressions respectively.^64^ These global reactivity descriptors provide valuable information regarding the evolution of molecular stability, charge-transfer capability, electrophilic behavior and overall chemical reactivity as the cluster size increases.^65^ Mulliken and Hirshfeld population analysis were also performed to further explore the electronic origin of intermolecular stabilization. The Mulliken population analysis describes the charge redistribution qualitatively, through partitioning of the electron density into overlapping atomic orbitals while Hirshfeld population analysis uses the stockholder partitioning scheme.^66,67^ The molecular electrostatic characteristics were further investigated through difference molecular electrostatic potential (ΔMEP) analyses which maps the redistribution of electrostatic potential due to aggregation.^68,69^ Finally, the thermal stability of the optimized aggregates was examined by using MD simulations in AMS with the DFTB engine.^70,71^ DFTB substantially cuts down computational expense of assessing the force value without losing the fundamental quantum mechanical description of chemical bonding.^72^ In-house Python scripts were also used to post-process this MD simulation while computing structural and dynamical descriptors of the simulated configurations. Numerical analysis was utilized by using scientific python packages, including NumPy for numerical calculations, SciPy for statistical analysis and Matplotlib for data visualization and the trajectory files generated by the AMS through PLAMS (Python Library for Automating Molecular Simulations). These analyses include the evaluation of relative shape anisotropy (*κ*^2^), Lindemann index (*δ*), orientational order parameter, inter-cage contact statistics, contact-angle distributions, contact survival autocorrelation functions, net cage charges and time-dependent covalent and intermolecular distance profiles. The reported quantities were directly calculated from the atomic coordinates and charges taken along the trajectories without intervention of an empirical fitting or the predictive model. All these simulations together give a complete and complementary theoretical picture for understanding the intermolecular interactions, charge redistribution and cooperative stabilization mechanism of the series of As_4_O_6_.

## 3. Result and discussion

The geometry of As_4_O_6_ cluster series was fully optimized using DFT in the ADF computational framework based on B3LYP-D3(BJ)/TZP level of theory. A detailed discussion of intra-cage rigidity, inter-cage interactions and cooperative stabilization effects is presented in the following sections.

### 3.1 Structural evolution analysis of C_*n*_ assembly

The structural and energetic evolution of C_1_–C_5_ cages assembly was systematically analyzed to elucidate whether the cluster evolution induces the covalent reorganization or influenced by the weak intermolecular forces. This section briefly includes the changes in structural parameters, size dependence of the bonding characteristics, inter-unit interactions and trends of stabilization which are discussed in detail below.

#### 3.1.1 Intra-cage interaction analysis

The As_4_O_6_ based cluster series are discrete cage units where the evolution of structure is controlled by the competition between inter-cage size-dependent interactions and rigid intra-cage covalent bonding. A detailed comparison of optimized and experimental parameters for the reliability and to quantify deviation from experimental values is presented in Table 1, whereas the optimized geometries of C_1_–C_5_ are shown in Fig. S1 and 1. The As–O bond lengths remain almost unchanged with different cluster sizes as listed in Table 1, the difference lies within 1.810–1.824 Å while the average bond length values change slightly from 1.814 Å to 1.818 Å. When compared with the experimental value, this corresponds to a very small deviation of approximately +0.031 Å for C_1_, which remains nearly constant throughout the series as illustrated in the Fig. 2d. The close proximity implies that the basic As–O bonding structure is well preserved in optimized structures and that the cluster evolution is not changing the intrinsic covalent bonding character in the cages.^73^ Further, distortion descriptors given by eqn (1) in Table 1, again support the structural stability. This can also be visualized in the Fig. 2d. The distortion index increases from 0.000 to 0.002 which is still very small and the standard deviation of As–O bond length calculated by eqn (2) is still within 0.000–0.004 Å. The quantitative measures clearly indicate that there is no significant deformation within the cages when they are assembled together.^74^ Angular parameters give further information about the structure as given in Fig. 1. The As–O–As angles are almost constant (128.4–128.8°) and close to the experimental value confirming rigidity of the bridging oxygen framework. The O–As–O angles are instead found to vary with size from 99.8° in smaller cluster to 100.3° in C_5_ with the structural adaptation found to be through angular flexibility, not bond elongation.^75^ C_3_ and C_4_ have values which lie in between the experimental reference (98.4°) which suggests that the angular response is not monotonic as the cluster size increases.^73^

**Table 1:** Intra-Cage and inter-cage Analysis giving structural and energetic comparison of As_4_O_6_ cluster series depicting size-dependent variations in geometry and stability

Structural parameters	C_1_	C_2_	C_3_	C_4_	C_5_	Exp. value
**Intra-cage analysis**						
Range of As–O bond lengths (Å)	1.817	1.815–1.821	1.810–1.818	1.812–1.819	1.812–1.824	1.786
Average As–O bond lengths (Å)	1.817	1.818	1.814	1.815	1.817	—
Standard deviation (*σ*) (Å)	0	0.002	0.001	0.001	0.004	—
Deviation from Exp. As–O (Å)	0.031	0.032	0.028	0.029	0.031	—
Distortion index (DI)	0	0.001	0.001	0.001	0.002	—
Bond angle analysis						
As–O–As	128.5°	128.4°	128.6°	128.6°	128.8°	128.7°
O–As–O	99.8	99.7°	99.7°	100.2°	100.3°	98.4°
As–As separation (Å)	3.244	3.249–3.259	3.232–3.244	3.232–3.242	3.236–3.270	3.20±0.05
O–O separation (Å)	2.779	2.762–3.927	2.761–3.943	2.766–3.933	2.766–3.927	

**Inter-cage analysis**						
Nearest O⋯O (Å)	—	3.052	3.429	3.425	3.047	3.022
Nearest As⋯As (Å)	—	3.970	4.058	4.129	3.973	—
Minimum As⋯O (Å)	—	3.048	3.571	4.127	3.043	—
Energy analysis (eV)						
Total bonding energy (*E*_C_*n*__)	–74.241	–149.306	–223.624	–297.997	–374.828	—
Energy per cage	—	–74.653	–74.541	–74.499	–74.966	—
Relative energy	—	–75.065	–149.383	–223.756	–300.587	—
Stabilization energy (total BE)	—	–0.824	–0.901	–1.033	–3.623	—
Binding energy (BE)/cage	—	–0.412	–0.300	–0.258	–0.725	—
Incremental energy	—	–0.824	–0.077	–0.132	–2.589	—

**Fig. 1:**
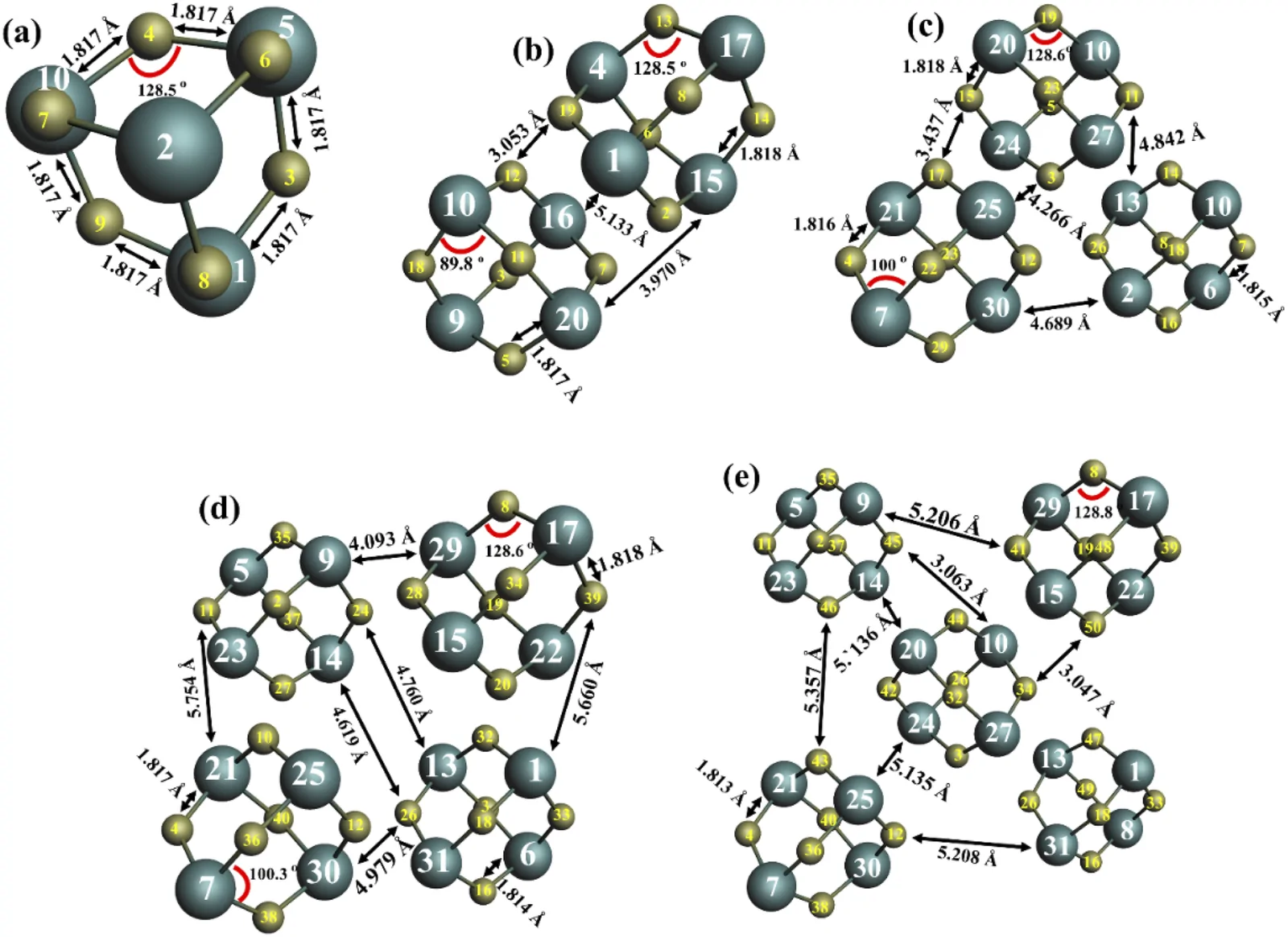
Intra-cage and inter-cage structural parameters of clusters (a) C_1_, (b) C_2_, (c) C_3_, (d) C_4_ and (e) C_5_ showing variations in bond lengths, bond angles and inter-cage distances.

**Fig. 2:**
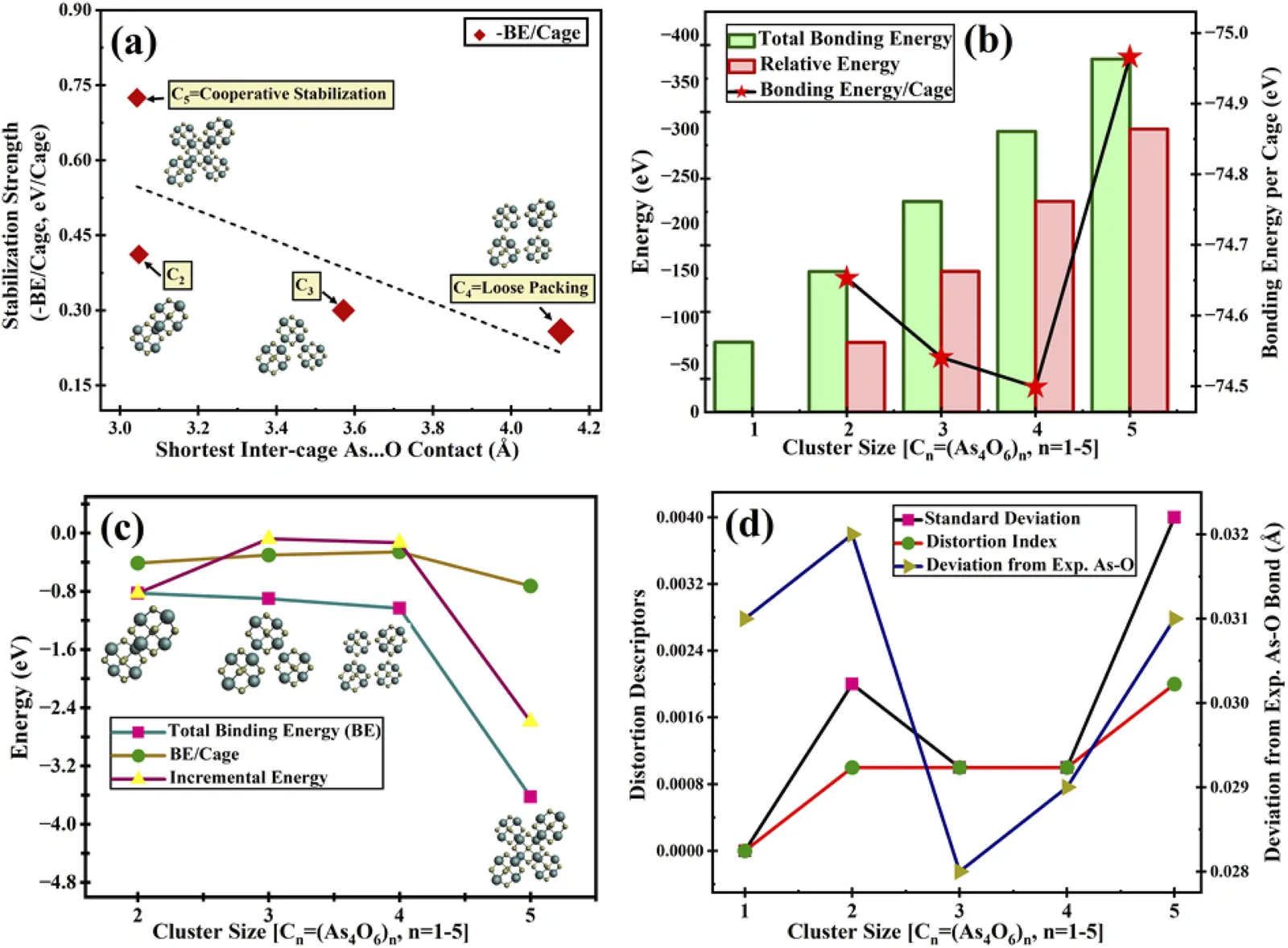
Size dependent evolution of structural stability and energetics for C_*n*_ cluster series illustrating (a) inter cage distance–stabilization relationship, (b) total and relative energy variation, (c) binding energy evolution including incremental and per-cage contribution and (d) distortion and deviation analysis.

The Optimized Geometries of the C_1_–C_5_ cages as illustrated in Fig. S1, shows all the structures along all axis with the side view revealing spatial arrangement of cages. This also suggests that the cage topology is preserved in all aggregation states and there is no apparent breakdown or rebuilding of the internal cage structure. This implies that rather than altering the bonding structure within the unit, clusters are formed by the assembly of intact units.^76^ These observations, from a physical point of view, create a clear distinction between the intra-cage and inter-cage effects. The intra-cage region exhibits strong As–O polar covalent interactions that impart rigidity to the cages, consistent with previous experimental and theoretical descriptions of cages.^26^ The deviations from the experimental value and the size-dependent variation are however, caused by inter-cage packing effects and not by covalent bonding changes.^77^ Moreover, the slight variations of angle provide direct information on the role of inter-cage interactions on the overall structural evolution of the C_*n*_ series.

#### 3.1.2 Inter-cage contact analysis

The inter-cage region of C_*n*_ is the primary region for determining the size dependent aggregation behavior with little invariant intra-cage framework.^78^ The interatomic distances listed in Table 1 and the structural variation shown in Fig. 1, give detailed behavior of cage–cage spatial organization as the cluster size is increased. The shortest inter-cage As⋯O distance reveals a clearly seen non-monotonic series variation. C_2_ is initially relatively compact with short inter-cage contacts of 3.048 Å indicating the incipient development of weak attractive interaction between the intact molecular units.^79^ This means that aggregation begins with a closeness of cages without breaking the covalent structure within.

The distance between cages starts to increase significantly for the clusters C_3_ and C_4_, whose values are equal to 3.571 Å and 4.127 Å respectively. This expansion reflects a modification of packing motif with a spatial arrangement based space-filling geometry which is derived from the lack of efficient packing. Hence, C_*n*_ series is particularly characterized by the fact that C_4_ configuration is a highly expanded one where the inter-cage interaction density and packing efficiency is reduced.^80^ This structural behavior is clearly visible in Fig. 1 where the intermediate clusters do not form a dense arrangement. The C_5_, by contrast, shows a return to a tight packing: the inter-cage distance is again 3.043 Å. In contrast, the coordination environment of C_5_ is higher than that of the C_2_ and it has multiple neighboring cage interactions which increases the interaction density. These structural features indicate that in bigger clusters, the stabilization is not a function of a single contact strength, but mediated by a combination of multiple weak inter-cage interactions.^81^ The overall conclusion from the inter-cage analysis is that the packing efficiency rather than the intra-cage deformation is dominant in the cluster series.

### 3.2 Thermodynamic feasibility of cluster evolution

The energetic stability of As_4_O_6_ offers a quantitative indicator for cage aggregation interactions as a function of size.^82^ The energetic parameters presented in Table 1, together with the trends shown in Fig. 2a–c, suggest that the stability depends nonlinearly on the cluster size.

The total bonding energy becomes more negative as the cluster size increases with the successive addition of As_4_O_6_ units. This number however is intrinsically size-dependent and does not directly measure intrinsic stabilization.^83^ The non-monotonic trend is more apparent in the binding energy per cage which was calculated by using eqn (4). The moderate stabilization of C_2_ and the decreased stabilization per cage of C_3_ and C_4_ are in line with the larger inter-cage separation distances and poorer packing efficiencies found in the structural analysis.^84^ The binding energy per cage of the C_5_ cluster is distinctly lower that of all the other clusters, indicating a significant increase in stabilization. The incremental energy values further support this enhancement, showing that the C_5_ is formed with a large energy gain compared to the preceding evolution steps, evaluated by eqn (3). The correlation in Fig. 2a confirms that generally lower As⋯O distances do lead to higher stabilization, however C_5_ does not follow the linear trend. This deviation indicates that the stability can be controlled by the number of weak inter-cage contact interactions rather than a single interaction between two cages in a compact arrangement.^85,86^ In summary, the energetic results indicate that the stabilization in the series of As_4_O_6_ clusters is determined not only by the cluster size, but also by the packing efficiency and the interaction density between the cages.

### 3.3 Electronic landscape evolution

The evolution of the electronic structure during As_4_O_6_ cage aggregation was first investigated through Total Density of States (TDOS) and the FMO states shown in Fig. S2 and S3 respectively.^87^ The TDOS profiles show that the spectral distributions of all aggregates are essentially the same for the C_1_–C_5_ series, suggesting that the electronic structure of As_4_O_6_ cage remains intact in the course of cluster evolution. The occupied electronic states are still mostly found in the range of about −12 to −6 eV as shown in the Fig. S2. Overall, the DOS intensity increases with the increase in cluster size due to increased number of interacting molecular orbitals. This behavior suggests that most of the aggregation is occurring by increasing the density of existing electronic states and not by the addition of electronic features.^88^ This also indicates that intermolecular forces involved in cluster evolution are relatively weak and are not able to change the intrinsic electronic structure of the individual cages. The partial DOS as shown in Fig. S3, provides greater insight into the origin of the frontier electronic states.^89^ The LUMO, in turn, is almost exclusively made up of As states whereas O is hardly present throughout the aggregation sequence.

Cages thus provides a relatively stable electronic building block for which the basic electronic properties are generally preserved during the aggregation process. Furthermore, the composition of HOMO and LUMO was determined by the ADF-SFO decomposition as summarized in Table S1. This information can be used directly to assess the change of orbital composition of these orbitals as individual cages approach one another and aggregates larger than single As_4_O_6_ cage.^90,91^ The orbital composition of the HOMO is decomposed indicating that it has a mixed As–O character during the aggregation sequence. The HOMO of the isolated cage (C_1_) has contributions from both As and O atoms; 47.99% and 48.92%, respectively, showing close to equal participation of both atoms. The percentages contributions may not add up to 100% because of the inter-atomic overlap populations that are part of Mulliken-type partitioning in the ADF-SFO analysis. During aggregation, the total contribution of As decreases from 47.99% to 43.80% while the contribution of O decreases more significantly from 48.92% to 31.67%. This is also confirmed by an examination of the percentage of the orbital-resolved populations: O is almost exclusively involved *via* its p orbital, as consistent with the predominant role of O lone-pair electrons. In contrast, the As contribution splits between both s and p orbital states which means that there is a hybrid orbital character. It is observed that the canonical Kohn–Sham orbitals are uniquely determined for each of the self-consistent field solutions. A unitary transformation acting on the subspace occupied by the orbitals does not alter the physical properties of the system like the total energy or the HL gap, but can lead to mixing of the orbital character of the near-degenerate frontier states. In the present As_4_O_6_ cluster series, the energy gap between the HOMO and HOMO−1 is small but not zero for all the clusters, suggesting a close manifold of frontier orbitals. The orbital-composition descriptors are therefore not presented as a quantitative result in this study, but as one piece among several complementary lines of evidence. In addition, independent charge and electrostatic analyses, which are not sensitive to orbital mixing effects, are used to complement the orbital-composition analysis.

A markedly different behavior is observed for LUMO as given in the Table S1. All of the As contribution comes from the p orbitals which is consistent with the fact that the frontier acceptor state is essentially a state of the p orbital of the As atom, for the whole aggregation sequence. The relative populations of these orbitals are conserved for the clusters in good agreement with the expected DOS analysis which shows maintained acceptor character of the LUMO in all cases. The orbital contributions listed in Table S1 allow to get a full picture but a set of quantitative orbital-composition descriptors that summarizes the main electronic properties of FMO in physically interpretable quantities.^92,93^ These descriptors are quantitative measures of frontier-state localization, arsenic s/p orbital balance, oxygen participation, LUMO orbital purity and HOMO orbital redistribution, allowing a systematic study of the electronic change upon the aggregation of cages. The mathematical definitions are given in SI file's Section 1.2 and the values calculated are summarized in Table 2, can also be visualized in the Fig. 3. The LUMO Localization Index is high during the aggregation process (73.70–91.00%) and this means that the frontier acceptor state is still mainly localized on the arsenic atom even in larger clusters. The temporary decrease seen for C_4_ is attributed to a small shift in the unoccupied electron density due to intermolecular interactions, while the recovery of the C_5_ shows that the As-centered acceptor state is still more favored when further aggregation takes place.^94^ The relative s/p orbital balance is improved from 0.29 (C_1_) to between 2.74 and 8.93 (larger aggregates), reflecting the progressive shift in this descriptor for HOMO. This is not an indication of the change in chemical bonding, but rather is an adaptation to the hybridization of the orbitals at the adjacent cages. The oxygen p-orbital participation also shows a decreasing trend which further supports that O lone-pair orbitals play decreasingly significant roles in building up the occupied frontier state consistent with the contribution role of O for HOMO. The LUMO p-orbital purity stays very high, even higher than 93.73% for the isolated cage and 100% for all the aggregated structures. This finding is consistent with the fact that the entire contribution of As to the LUMO is from p orbitals thus indicating that the electronic properties of the frontier acceptor state is almost unaltered by aggregation.^95^ These trends in the evolution of both SFO-based descriptors suggest a consistent reorganization of the electronic structure on aggregation, with the reduction of Oxygen p-orbital participation corresponding to a loss of oxygen lone-pair contribution and the increase of Relative s/p obital balance compensating for that loss to maintain the overall structure. The near-constant LUMO p-orbital purity indicates that these changes occur only in the occupied states while the LUMO is largely preserved. Overall, these results indicate that aggregation alters the distribution of FMO but maintains the intrinsic electronic nature of As_4_O_6_ cage which points its potential as a stable electronic building block of larger arsenolite structures.

**Table 2:** Quantitative analysis of the orbital-composition descriptors (%) based on the SFO, illustrating the comparative metric for physical interpretation of cluster aggregation

Descriptors	C_1_	C_2_	C_3_	C_4_	C_5_
LUMO localization index	91.00	86.00	84.41	73.70	86.85
Relative s/p orbital balance	0.29	6.17	2.74	8.93	5.56
Oxygen p-orbital participation	48.92	33.18	39.39	32.09	31.67
LUMO p-orbital purity	93.73	100	100	100	100

**Fig. 3:**
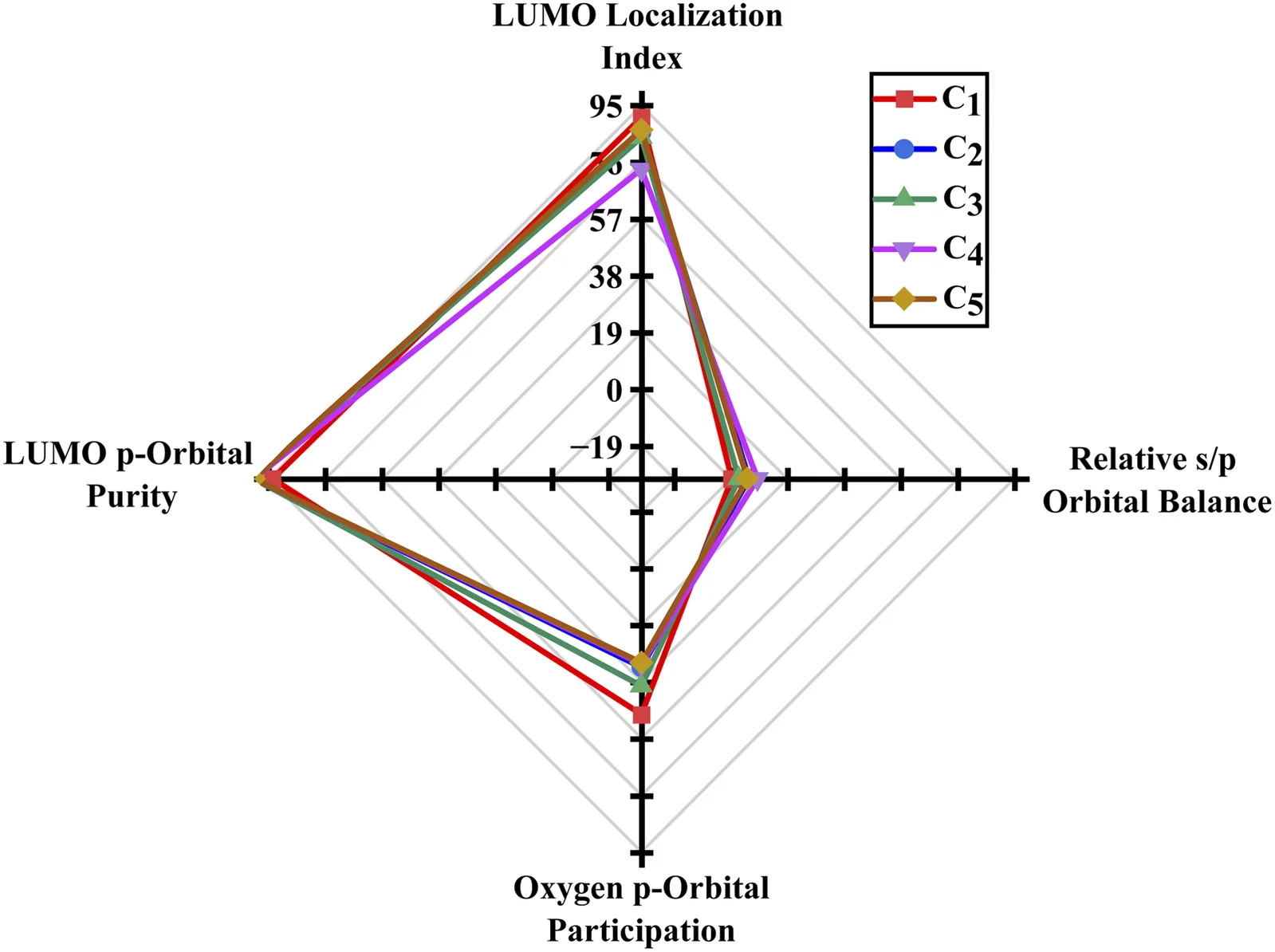
Evolution trends of the orbital-composition descriptors as a function of the cluster size illustrating the physical interpretation of the SFO analysis.

#### 3.3.1 Global reactivity descriptors

The evolution of the FMO provides further insight into the electronic consequences as HOMO and LUMO isosurfaces show a progressive spatial redistribution with the increasing number of interacting cages as shown in Fig. S4.^96^ The orbital energies and the conceptual DFT descriptors are summarized in Table S2. The computed HL gap gradually decreases from C_1_ to C_4_ and then increases slightly for C_5_. This behavior indicates that the early steps in aggregation increase the electronic flexibility to redistribute the charge. The partial recovery of HL gap in C_5_ indicates that incorporation of the central cage does not continue this trend indefinitely as shown in Fig. 5. As noted, the electronic gap calculated here for the isolated cluster (HL gap of 6.158 eV for C_1_) is significantly different from the reported gap for bulk crystalline As_4_O_6_ from previous DFT studies, where the electronic gap was deduced from TDOS calculations (1.5 eV (PBE) and 2.3 eV (B3LYP)). This difference is due to the different electronic-structure frameworks employed in the two approaches. In the previous work, the electronic states were delocalized over a periodic crystal while the present work considers a finite cluster (As_4_O_6_)_*n*_ with its discrete molecular orbitals and without periodic boundary conditions.^28^ The electronic gap in the present case then is not a band gap from periodic electronic states, but a gap between the HOMO and LUMO energy levels. This trend is further reflected by the global hardness (*η*) and softness (*S*). The softness of the compounds increases from C_1_ to C_4_ as the chemical hardness decreases which indicates that the intermolecular aggregation increasingly allows electronic polarization and charge redistribution as trend is shown in Fig. 4c and d. Such behavior is expected as increasing intermolecular interactions provide additional pathways for electronic communication among neighboring cages.^97^

**Fig. 4:**
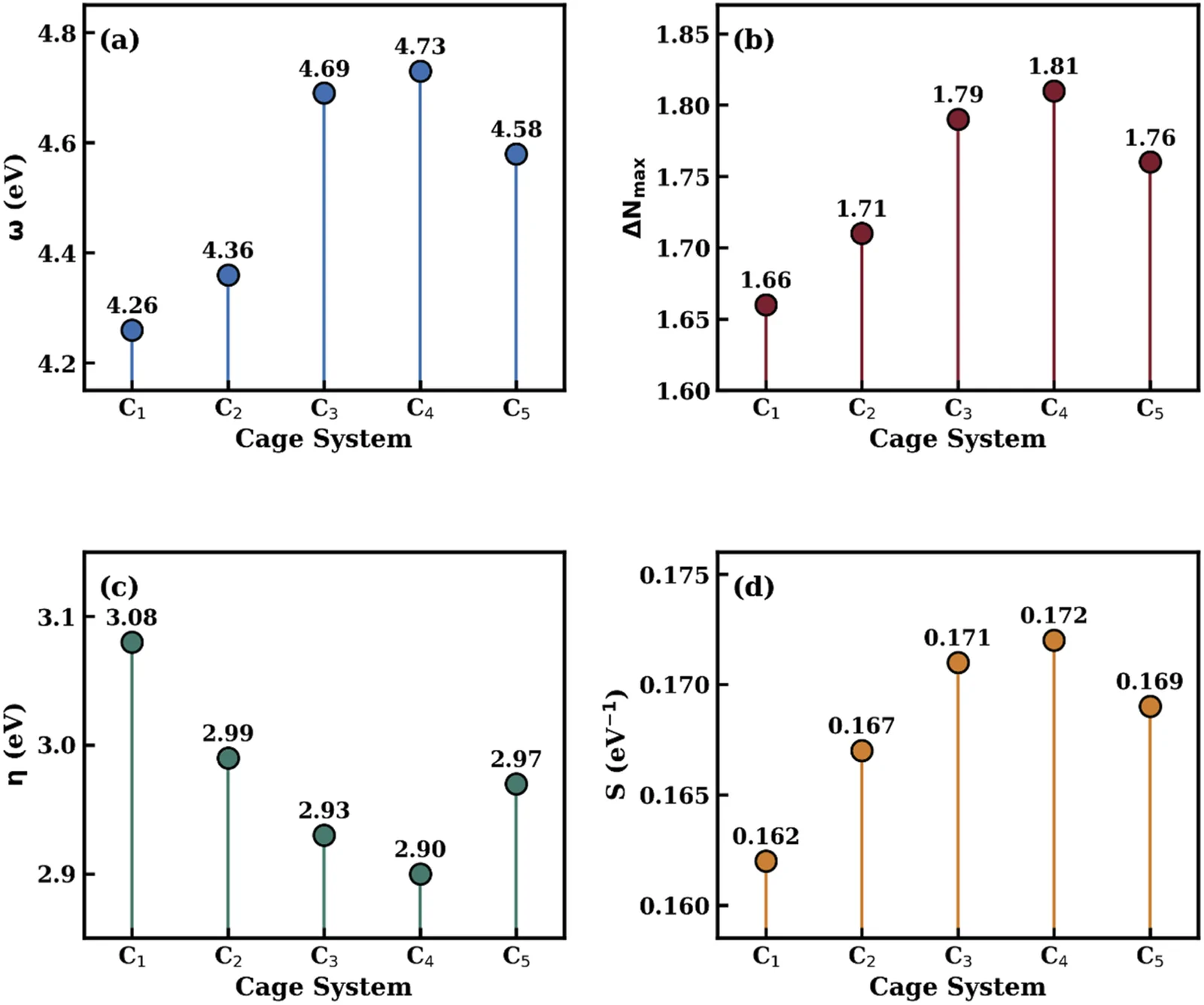
Evolution of the global reactivity descriptors calculated for the As_4_O_6_ cluster series as a function of cluster size: (a) electrophilicity index (*ω*), (b) maximum electronic charge acceptance (Δ*N*_max_), (c) global hardness (*η*) and (d) global softness (*S*).

The small increase in hardness and the corresponding decrease in softness seen in C_5_, however, suggests that the electronic structure is partly stiffened. A similar tendency is observed for the electrophilicity index (*ω*) which increases continuously during the early stages of aggregation and reaches its maximum for C_4_ before decreasing slightly in C_5_ as shown in Fig. 4a. This evolution suggests that the clusters can first accommodate electronic perturbations better. Additionally, the cooperative electrostatic network refers to a non-additive electrostatic regime in which the electrostatic potential of the cluster is not simply the sum of the electrostatic potential of its isolated cages but is affected by the total inter-cage collective interactions. However, once the cooperative electrostatic network is fully formed in the C_5_, further charge redistribution is not as energetically favorable.^25,98^ This is supported by the frontier orbital distributions which are displayed in Fig. 5 and S4. The FMO of larger aggregates are increasingly delocalized over a number of cages, as compared with the localized orbital distributions of the smaller clusters, indicating increased intermolecular electronic communication as clusters grow.^99^ The maximum electronic charge acceptance (Δ*N*_max_) exhibits a trend similar to that of the electrophilicity index as shown in Fig. 4b, indicating that the ability of the clusters to accommodate additional electronic charge initially increases during aggregation. This behavior shows the gradual increase of intermolecular electronic interaction with the progressive coupling of the neighboring cages.^100^

**Fig. 5:**
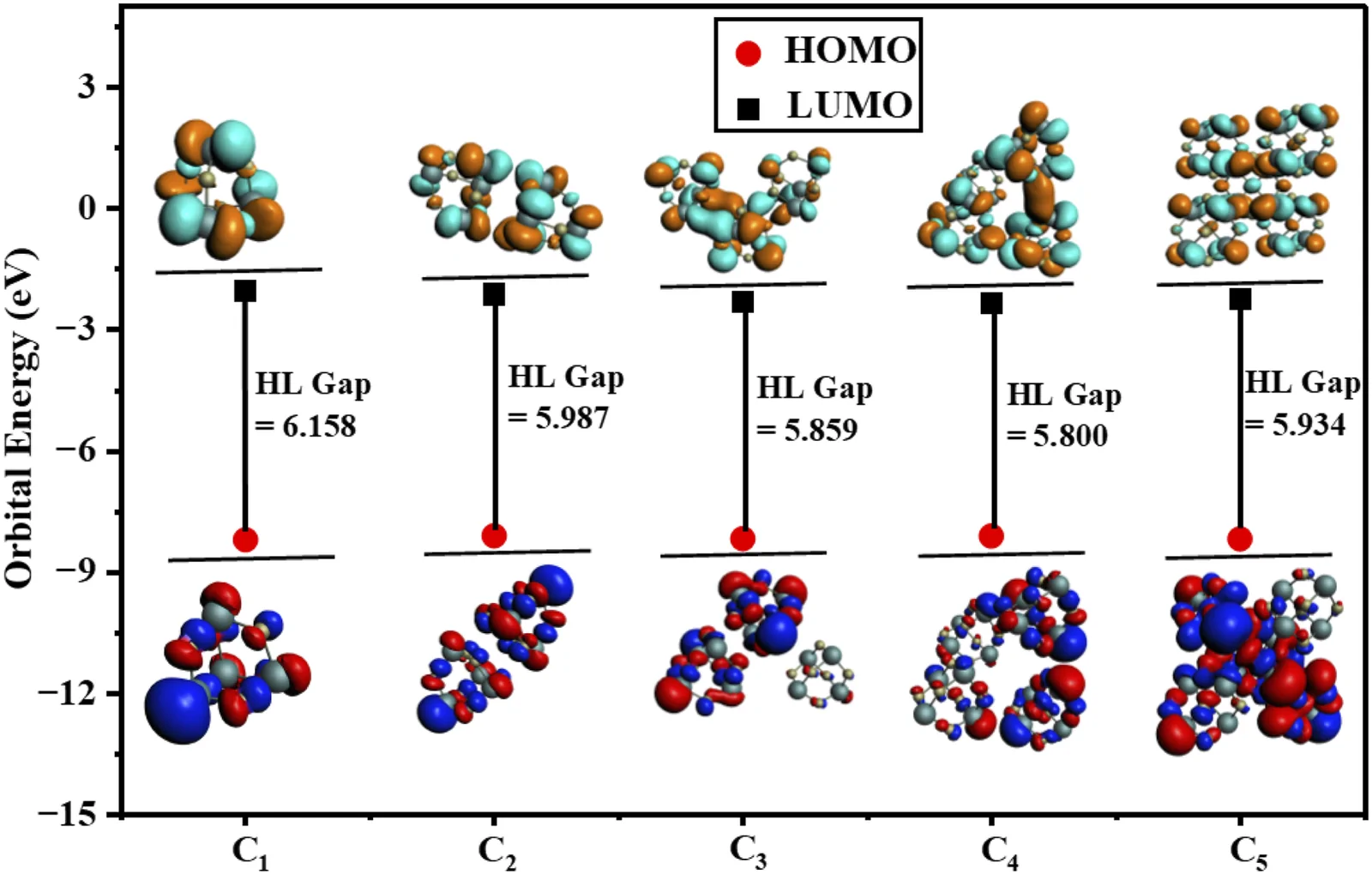
FMO energy-level diagram with HOMO and LUMO isosurfaces of the C_1_–C_5_ series, to give a comparative visualization of the frontier electronic structure at the HL energy gap.

The slight decrease observed for C_5_ suggests that cooperative electronic network reaches a balanced configuration after incorporation of central cage. As a result, while thermodynamically most stable aggregate is C_5_ but this electronic structure is less sensitive to additional charge redistribution.^101^ This size-dependent evolution of electronic properties may be used as a guideline for the design of electronically tunable cage-based molecular assemblies, molecular crystal engineering and supramolecular electronic materials.

#### 3.3.2 Molecular multipole moments

The computed molecular dipole and quadrupole moments of the cage aggregates of As_4_O_6_ are listed in Table S3. The dipole moment of the cluster increases from 0.002 D for C_1_ to 0.165 D and 0.235 D for C_2_ and C_3_ respectively and then decreases rapidly for C_4_ and C_5_. The quadrupole moments also show the same trend with the components of C_4_ and especially C_5_ being much smaller while C_3_ gives deviation from electrostatic symmetry. The dipole and quadrupole moments increase from C_1_ to C_3_, suggesting that electronic redistribution is important when cages begin to aggregate together with the onset of intermolecular interactions.^102^ The electrostatic polarization and anisotropic charge distribution of the assembly is still developing during this evolution stage, leading to a more favorable packing arrangement.^103^ The well-defined multipole moments measured for C_3_ are thus an intermediate state in the cluster evolution where cooperative interactions are still developing. Despite of intermolecular interactions and energetic stability analysis, both C_4_ and C_5_ possess significantly smaller dipole and quadrupole moments. In keeping with this structural arrangement, C_5_ has one of the smallest dipole moments with almost vanishing quadrupole moment components, meaning the balanced redistribution of electron density.^104^ This apparent decrease in dipole moment is not due to a decrease in polarization, but due to geometric cancellation. C_5_ shows the largest charge separation in the series (Δ*q* = 2.381 e (Section 3.4.2, Fig. 9)), but the charge separation is spread more or less symmetrically over the four peripheral cages around the central cage resulting in partial cancellation of the local polarization vectors in the global multipole moment. It leads to a small net dipole moment in spite of an improved local charge separation and corresponds to a characteristic “balanced redistribution” of the electron density in the C_5_ cluster.

### 3.4 Charge distribution and electrostatic response

To gain further insight into the electronic origin of cooperative stabilization, the redistribution of electronic charge and the evolution of electrostatic environment were systematically investigated by ΔMEP analysis, Mulliken and Hirshfeld charges as discussed below.

#### 3.4.1 Molecular electrostatic maps

To elucidate the electronic origin of progressive stabilization observed during As_4_O_6_ cluster evolution, ΔMEP was analyzed using complementary 3D isosurfaces visualizations and ΔMEP profiles as shown in the Fig. 6, 7 and 8. In the Fig. 6, The electron-density and ΔMEP isosurfaces were generated with isovalues of *ρ* = 0.05 a.u. and ΔMEP = ±0.005 a.u. respectively with positive and negative ΔMEP regions as plotted in blue and red respectively. The distance axis in the Fig. 6 represents the linear distance from cage to cage. Panel (a) is the intramolecular ΔMEP profile of isolated C_1_ while panels (b–d) depict intermolecular cage–pair interactions. The curve labels indicate certain interacting cage pairs as highlighted by different colors peaks. These representations give a coherent description of the stepwise transfer of electrostatic potential during evolution.^105,106^ In the Fig. 7a and b, for C_1_ the electrostatic distribution is relatively localized, with small perturbations along the selected pathway as shown in Fig. 6a. It is suggesting that electrostatic field is mainly determined by the intrinsic charge distribution of a single cage. When C_2_ is formed as in Fig. 7c and d, ΔMEP isosurfaces reaches into intermolecular contact region and mapped electron-density surface reveals that a continuous electrostatic interface forms between cages. This behavior is characterized by the appearance of a broad ΔMEP maximum in line profile as shown in Fig. 6b, which is sign of onset of intermolecular electronic communication. The electrostatic complexity is additionally elevated in C_3_ where ΔMEP and density-mapped electrostatic isosurfaces crosses multiple cage contacts and creates multiple electrostatic domains, as shown in Fig. 7e and f. In Fig. 6c, the three pathways have a non-equivalent ΔMEP profile, with different amplitude and peak position, indicating different local electrostatic environments for each cage.^107^

**Fig. 6:**
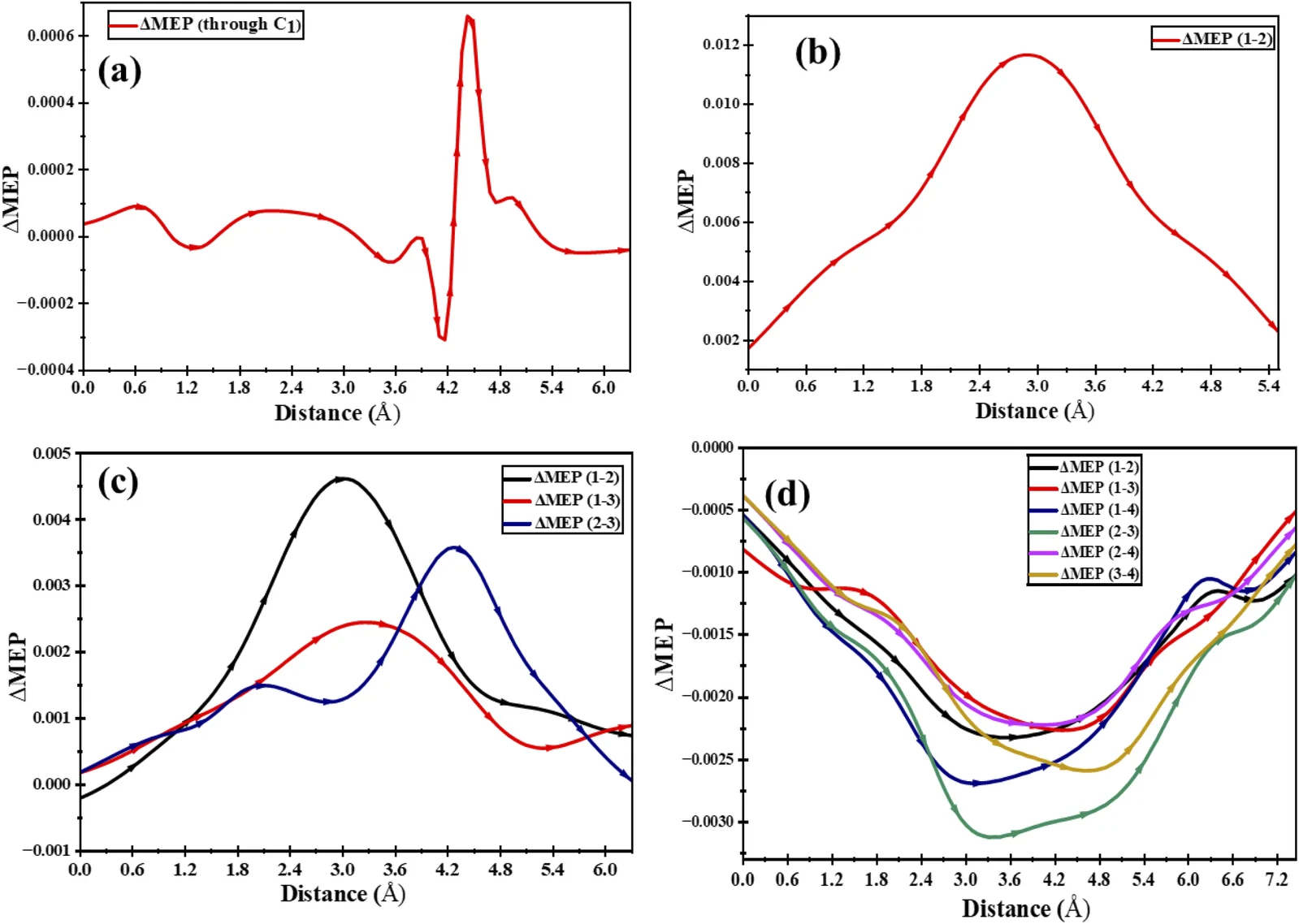
ΔMEP profiles along the different intermolecular pathways in the cluster series As_4_O_6_. Panels (a–d) correspond to the C_1_, C_2_, C_3_ and C_4_ clusters respectively.

**Fig. 7:**
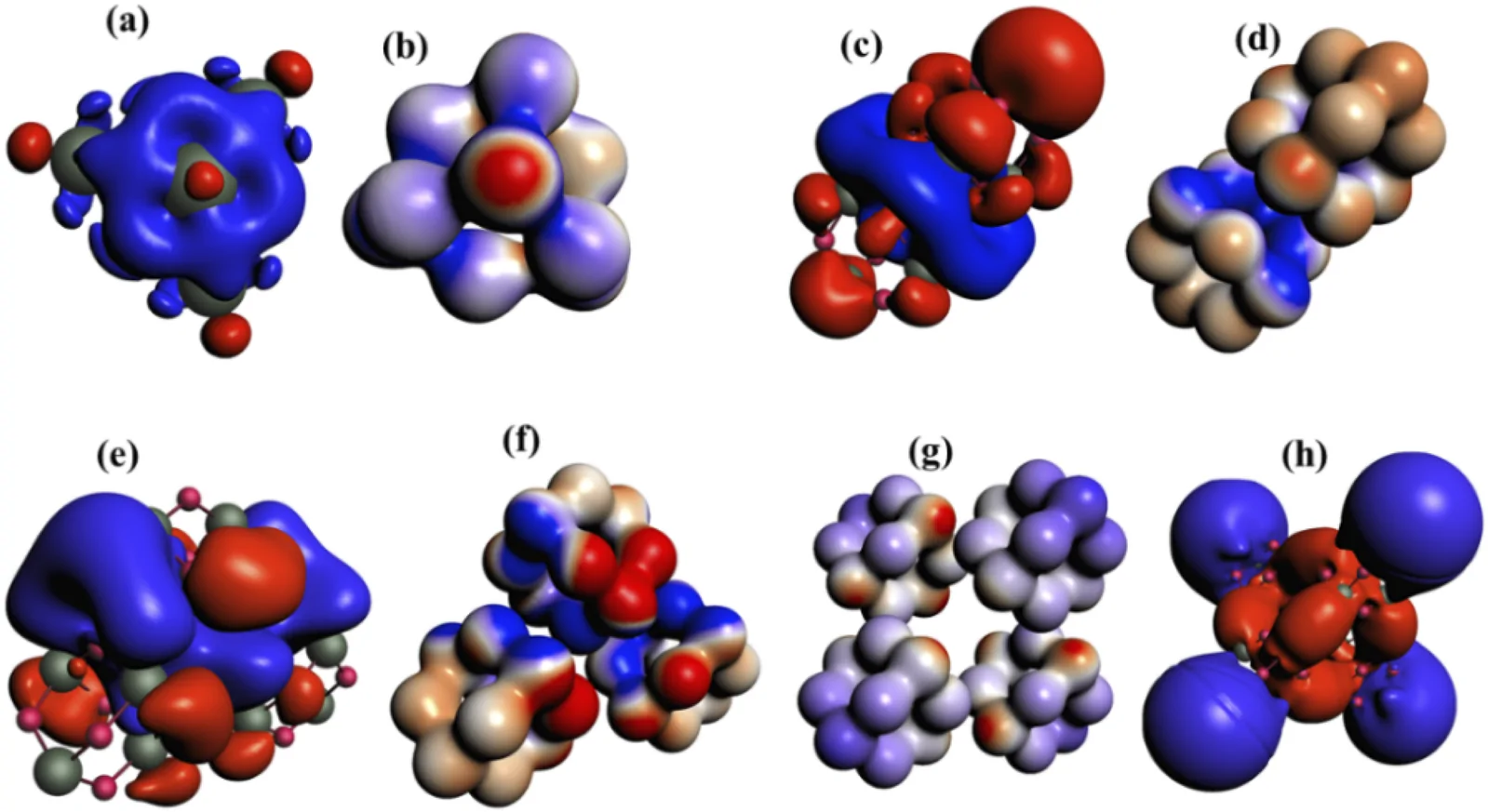
Evolution of the electrostatic environment during the evolution of As_4_O_6_ clusters. The electron-density isosurfaces are shown in panels (a, c, e, g) while the ΔMEP isosurfaces are plotted in panels (b, d, f, h) for the C_1_–C_4_ clusters.

**Fig. 8:**
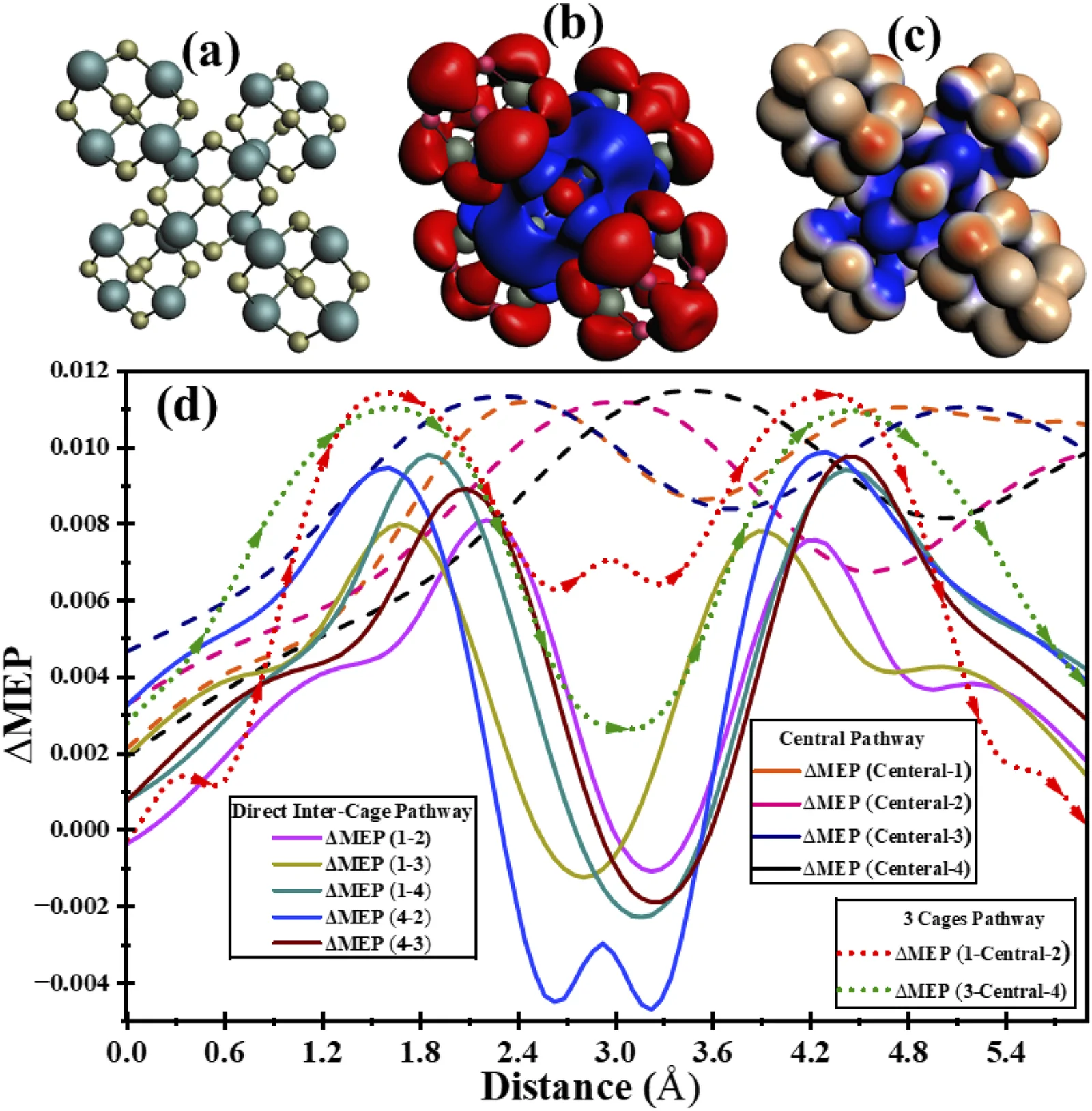
Electronic characterization of the C_5_ cluster. (a) Optimized geometry, (b) ΔMEP isosurfaces, (c) electron-density isosurfaces mapped with the ΔMEP and (d) ΔMEP profiles calculated along the direct inter-cages and the central cage pathways, showing the evolution of electrostatic redistribution associated with the central cage and the surrounding cages.

These observations reveal not only pairwise interactions but also suggest the development of an interconnected electrostatic network in the early stages of the cluster evolution. This is because the formation of C_4_ leads to a more homogeneous electrostatic distribution and surfaces of ΔMEP that are more regular and continuous over the aggregate as depicted in Fig. 7g and h.

The electron-density is projected and appears more even distribution of positive and negative electrostatic regions. In contrast, the ΔMEP profiles of C_4_ are predominantly negative ranging from about −0.5 × 10^−3^ to −3.2 × 10^−3^ a.u. along the 6 different intermolecular paths. The reduced variation in the pathways however, indicates that electrostatic perturbations are not limited to a pair of cages but distributed across the cluster.^108^ This role of cooperative electrostatic redistribution is even more evident in the C_5_, in Fig. 8.

C_5_ has direct peripheral pathways and pathways through the central cage, unlike C_2_–C_4_. The profiles of the ΔMEP in the central pathways are smoother and more extended as in Fig. 8d while the corresponding ΔMEP isosurfaces as in (Fig. 8b and c), depict an interconnected electrostatic framework. The central cage thus serves as an electrostatic mediator, transferring the electrostatic potential from the central cage to the surrounding cages. Overall, the three complementary representations provide a coherent description of the aggregation process.^109^ The sequential evolution of the ΔMEP distribution offers direct evidence of electronic changes in system which gives electronic support to cooperative stabilization.

#### 3.4.2 Charge redistribution analysis

A systematic evolution of electronic polarization in the cluster series C_*n*_ = (As_4_O_6_)_*n*_, *n* = 1–5 is identified by Mulliken population analysis which shows that the electronic polarization is determined by the interplay between the intra-cage bonding and the inter-cage interactions.^110^ The As_4_O_6_ unit has an intrinsically polar covalent As–O framework: in accordance with the electronegativity difference between As (2.18) and O (3.44).^111^ The characteristic electronic polarization is largely retained throughout C_1_–C_4_ chains which means that the covalent structure of the cage units is preserved inside.^112^ The optimized geometries for all the clusters shown in Fig. S5a have confirmed that the cage structure is unchanged for C_1_–C_4_ clusters. But for C_5_ cluster, a clear spatial electronic heterogeneity shows up, with the Mulliken charge being much more localized in central cage. This behavior is quantitatively confirmed by Mulliken charge data summarized in Fig. S5b: relatively small variations in charge separation (Δ*q* = 2.148–2.211 e) are observed for C_1_–C_4_ clusters while a significant increase is observed for C_5_ (Δ*q* = 2.381 e).

The cooperative aspect of electronic polarization caused by aggregation is evidenced by simultaneous improvement of the electron-rich oxygen centers and electron-poor As centers.^113^ The main advantage at higher aggregations is that inter-cage interactions become spatially non-uniform leading to the formation of a local electronic polarization hotspot at the cluster center.^114^ Further statistical information is provided in Fig. 9a in which the Mulliken charge histograms display a clear enhancement in the charge distribution for C_5_. This spreading is explained by an increase in electronic heterogeneity which is caused by an improvement of the electrostatic coupling between cages. This cooperative electronic restructuring can also be quantitatively illustrated in Fig. 9b showing the deviation Δ*q* from the C_1_–C_4_ baseline as a function of the cluster size and the corresponding percentage change. The non-monotonic growth of C_1_–C_4_ is followed by a sharp positive deviation at C_5_ (+9.24%) that signals a change of electronic and cooperative polarization regimes. Additionally, the electronic polarization variation is consistent but moderately different for all cluster sizes (C_1_–C_5_) as obtained by Hirshfeld charge analysis. The Hirshfeld charges are restricted to a small range (around +0.50 to −0.34 e), suggesting that the intrinsic As–O polar-covalent framework is preserved to a large degree during aggregation. However, a more pronounced cluster-size dependence is observed in Mulliken, which is a consequence of the difference between the two partitioning schemes used: Hirshfeld analysis partitions the electron density based on the isolated atomic density, whereas Mulliken analysis partitions the electron density based on the molecular orbital population.^115^ Both methods consistently identify C_5_ as the cluster with the highest degree of electronic polarization. Physically, which is due to electrostatic reinforcement from the confinement which enables the maximum polarization behavior for the central cage from the surrounding units.^116,117^ The structural integrity of the cages of As_4_O_6_ is confirmed by synchrotron X-ray diffraction studies up to ∼30 GPa and the stereo chemically reactive lone electron pairs of As(iii) are still pointing outward from the cage framework. These lone pairs play a role in the host-guest charge redistribution in H_2_-loaded arsenolite systems with a Mulliken population analysis showing a partial transfer of the electrons from As centers to the guest molecules under compression.^118^ In current finite-cluster model, the polarization and packing of C_5_ are found to be enhanced which signifies the cooperative electronic effects even without external pressure.

**Fig. 9:**
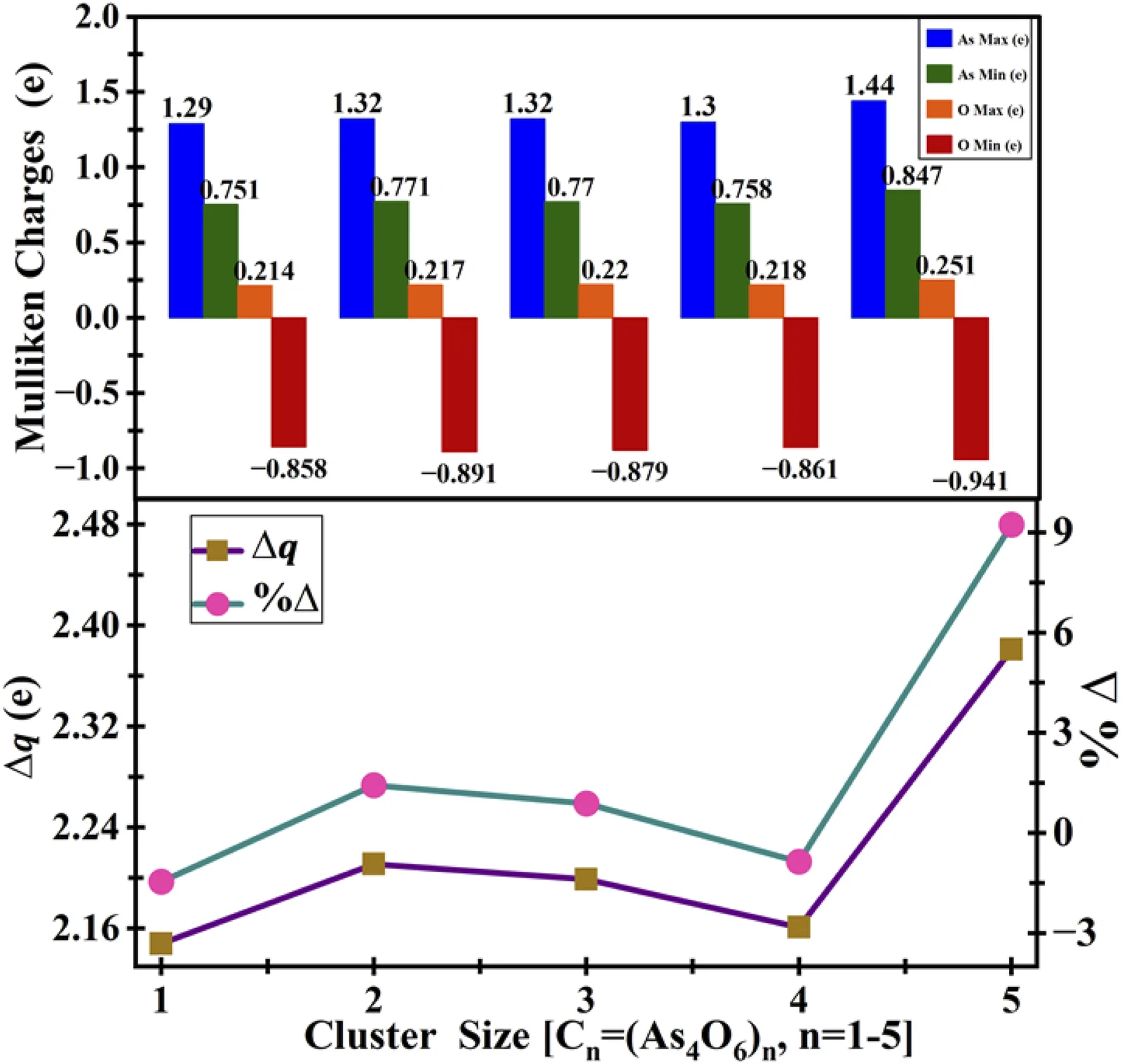
(a) The histogram plots of Mulliken charge distribution for (As_4_O_6_)_*n*_ demonstrating electronic heterogeneity as a function of size. (b) Charge separation, Δ*q* and percent deviation from C_1_–C_4_ baseline.

### 3.5 Thermal stability analysis

The thermal stability and dynamical behavior of the As_4_O_6_ cluster series was further assessed using DFTB-based MD simulations and discussed below.

#### 3.5.1 Temperature induced energy fluctuations

The thermal stability of the As_4_O_6_ cluster series (C_1_–C_5_) was evaluated by DFTB-based MD simulations at 300 and 500 K temperature. Fig. S6 shows the energy and temperature trajectory of each cluster during the simulation with all the clusters showing stable energy profiles and small variations around the equilibrium values at 300 K. There is no systematic drift or sudden changes in energy which shows that the optimized assemblies are dynamically stable in ambient temperature.^119^ Similarly, the temperature profiles fluctuate around the target temperature with normal statistical variations, confirming proper thermal equilibration of the system as shown in Fig. S6. As the temperature is raised from 300 K to 500 K, the amplitude of the energy and temperature fluctuations both increase as would be expected with an increase in temperature as illustrated in Fig. S7. However, the energy paths are well balanced and do not show any gradual shift or abrupt jumps and the temperature is also kept around the desired range throughout the simulation.^120^ From these observations it is concluded that the cluster assemblies are not structurally unstable or permanently deformed by the extra thermal motion. Overall, the MD simulations demonstrate that the dynamic stability of As_4_O_6_ cluster series under both ambient and elevated thermal conditions.

#### 3.5.2 Trajectory based structural and dynamical analysis

MD trajectories were then investigated using the structural and dynamical descriptors, to determine if the trends predicted by the static DFT calculations remain at finite temperatures.^121^ These descriptors offer complementary information about the evolution of molecular packing, thermal mobility, orientational organization, contact geometry, contact persistence, cage contact density and cage polarization.^122^ The shape anisotropy (*κ*^2^), the Lindemann index (*δ*) and the orientational order parameter (*S*) of C_*n*_ cluster series at 300 and 500 K are summarized in Fig. 10. The *κ*^2^ values demonstrates a clear structural evolution with increasing aggregation as shown in Fig. 10a. The C_2_ and C_3_ have relatively large anisotropy values (*κ*^2^ ≈ 0.321–0.355 at 0 K) which are characteristic of elongated and chain-like geometries. In contrast, the value of *κ*^2^ drops significantly for the C_4_ and tends to zero for the C_5_, suggesting the formation of compact and almost isotropic molecular assemblies. Thermal fluctuations at 300 K and 500 K lead to a slight increase of *κ*^2^ for all clusters but do not affect the ordering of trend; C_5_ is the cluster with minimum anisotropy value within the range of investigated temperatures. The reduction in *κ*^2^ is thus not only a result of the geometric compactness but also a result of formation of a very well-coordinated intermolecular packing network typical of cooperative stabilization.^123^ The Lindemann index (*δ*) is a ratio of the amplitude of atomic displacement and serves as a good criterion for distinguishing the rigid from highly mobile molecular assemblies as displayed in Fig. 10b. At 300 K, both the C_2_ and the C_5_ remain solid-like and have δ′s close to 0.024 while having highly restricted atomic motion. In contrast the C_3_ and C_4_ have significantly larger Lindemann indices (*δ* ≈ 0.113–0.125) that approach or exceed the conventional threshold for liquid-like dynamical behavior.^124^ Increasing the temperature to 500 K enhances thermal motion in all clusters, but the relative stability sequence remains essentially unchanged. With the higher thermal energy, C_5_ still has relatively small displacement amplitudes (*δ* ≈ 0.121); however, the C_4_ shows the highest increase in atomic mobility (*δ* ≈ 0.224). These observations show that thermal fluctuations can be accommodated in different ways depending on the intermolecular topology.^125^ The interlocked packing motif in C_5_ due to several intermolecular contacts, decreases the atomic mobility and maintains the rigidity of whole packing. The orientational order parameter further supports this explanation by studying the dynamics of rotations of neighboring cages in the simulation as show in Fig. 10c.

**Fig. 10:**
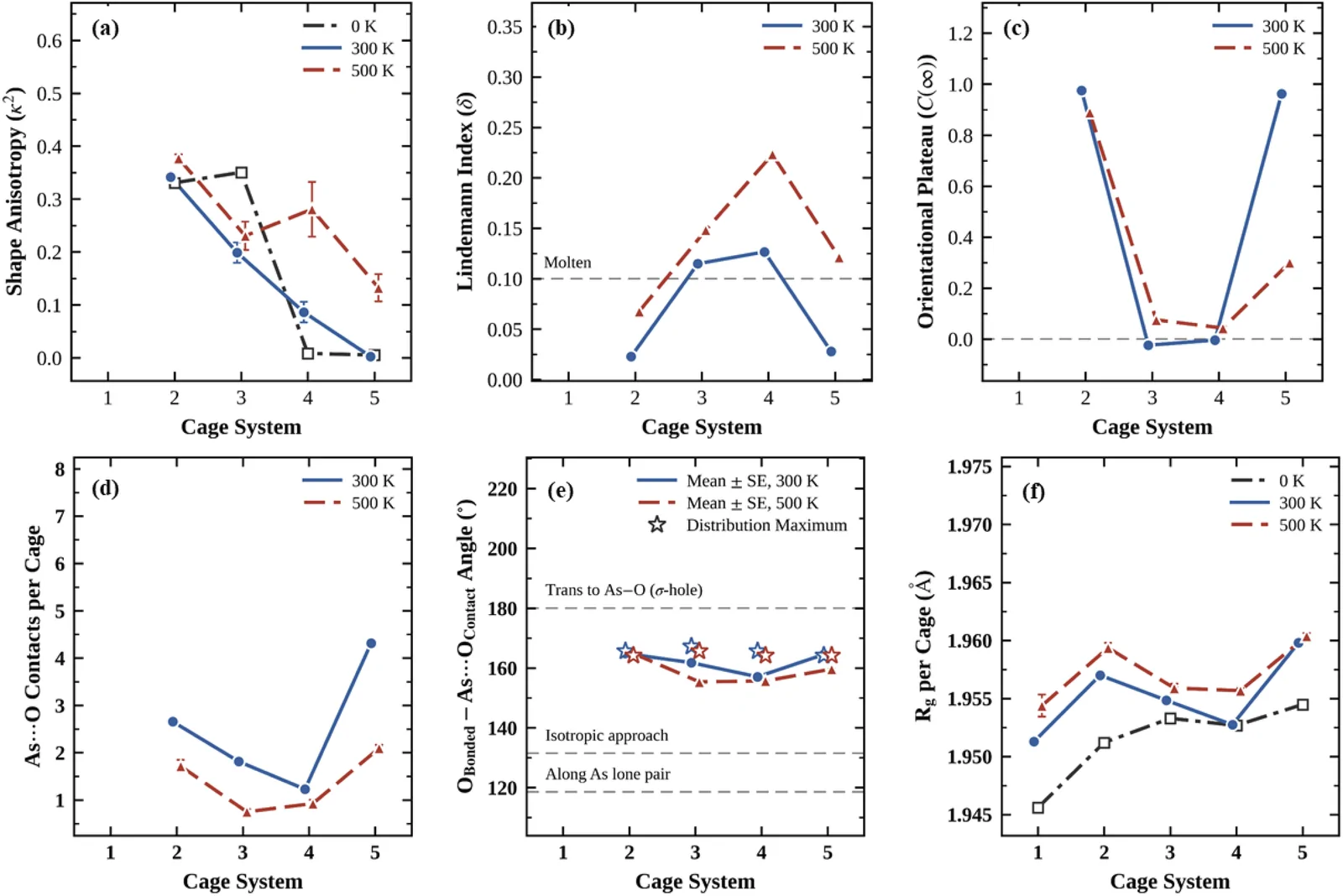
Evolution of structural and dynamical properties of (As_4_O_6_)_*n*_ clusters (*n* = 1–5) at 300 and 500 K: (a) relative shape anisotropy (*κ*^2^), (b) Lindemann index (*δ*), (c) orientational order plateau, (d) contacts per cage, (e) O_Bonded_–As⋯O_Contact_ angle and (f) radius of gyration per cage (*R*_g_).

Both C_2_ and C_5_ show almost complete orientational correlation with the time order parameters approaching unity at 300 K, meaning that the relative molecular orientations are essentially fixed along trajectory.^126^ In contrast, C_3_ and C_4_ quickly lose orientational correlation whose order parameter will approach zero, reflecting the unrestricted rotation of neighboring cages. The orientational correlations are certainly less strong in all systems upon increasing the temperature to 500 K. The C_2_ has the highest orientational order while the C_5_ has a significant amount (≈0.300) well above the values found for C_3_ and C_4_ which are essentially disordered.^127^ This also shows no sign of breaking down even at partial thermal relaxation, while the Lindemann index and *κ*^2^ provide a detailed picture of the increased vibrational amplitude in this region. Importantly, these descriptors also explain the higher stability of the C_5_, physically. Inter-cage contact density, contact geometry, contact persistence and cage polarization descriptors directly focus on the change in the network of intermolecular interactions.^128^ The average inter-cage contact density illustrated in Fig. 10d, shows a significant non-monotonic relation with cluster size. At 300 K, the number of contacts decreases from 2.7 contacts per cage in C_2_ to 1.8 in C_3_ and reaches a minimum of 1.2 in C_4_, before increasing sharply to 4.3 contacts per cage in C_5_. The absolute contact population decreases by around 40–50% upon increasing the temperature to 500 K but the structural hierarchy does not change. The corresponding contact-angle distributions as illustrated in Fig. 10e, further demonstrate that these As⋯O interactions are highly directional. The average O_Bonded_–As⋯O_Contact_ angles remain between 155° and 168°, significantly exceeding the isotropic limit and approaching the trans direction of the As–O bond. The mean angles of C_2_ and C_5_ are the largest while mean angles of C_4_ are smaller (∼158°) and distributions are broader, showing that intermolecular interactions are not as organized as in C_2_ and C_5_.^129^

The radius of gyration per cage (*R*_g_) is a direct measure of structural integrity of As_4_O_6_ unit in terms of aggregation state and temperature as given in Fig. 10f. Across the cluster series, *R*_g_ remains confined within a narrow range of ∼1.945–1.960 Å with variations below 0.015 Å at 0, 300 and 500 K. The near constancy implies that the intrinsic geometry of the As_4_O_6_ cage is preserved and that only a modest thermal expansion (∼0.01 Å) occurs at high temperature due to thermal vibration rather than cage distortion.^130^ The dynamic stability of contact network was evaluated through the contact autocorrelation function *C*(*t*) as illustrated in Fig. 11a and b. The time plateaus (∼0.882 and ∼0.901) are observed for C_2_ and C_5_ at 300 K where intermolecular contacts are present for a long time as shown in Fig. 11a. In contrast, C_3_ retains only weak residual correlations (∼0.183), whereas C_4_ loses almost all contacts within the first 2 ps. Contact lifetimes drop for all aggregates at 500 K and C_5_ ends up with contact correlations that are still measurable after 8 ps as shown in Fig. 11b. This outcome shows that the thermal stability of the cooperative network of C_5_ is significantly higher than that of smaller networks.^131^ The net cage charge distributions given in Fig. 11c and d are electronic consequences of this cooperative organization. At 300 K, the C_1_–C_4_ are essentially neutral, while the C_5_ has a charge separation that assigns the charge of about +0.040 e to the central cage and the approximately −0.01 e to each of the four surrounding cages. This collective polarization disappears almost completely at 500 K where all cages become nearly charge neutral as shown in Fig. 11d. This temperature dependence suggests that the observed charge redistribution becomes more stable in the compact interlocked geometry of the C_5_ and that the cooperative intermolecular organization is gradually reduced as the thermal motion reduces the intermolecular organization.^132^ This mechanism is further supported by the time dependent trajectories of the structures in Fig. S8 and S9. As shown in Fig. S8, the intra-cage As–O bond lengths remain nearly constant for all aggregates at both temperatures, confirming that the molecular cages preserve their structural integrity throughout the simulations. In contrast, the inter-cage contact trajectories given in Fig. S9 show pronounced differences among the aggregates. C_5_ has about 22 simultaneous contacts at 300 K, without significant fluctuations, while C_3_ and C_4_ have significantly fewer and more variable contacts. The contact population of all systems is reduced at 500 K, but the C_5_ has a much higher contact number than the other aggregates. This is because C_5_ has a more stable residual contact network than the C_2_–C_4_. A good correspondence with these dynamical descriptors and with static DFT analyses provides a common picture of the mechanism for stabilization of arsenolite clusters at finite temperature.

**Fig. 11:**
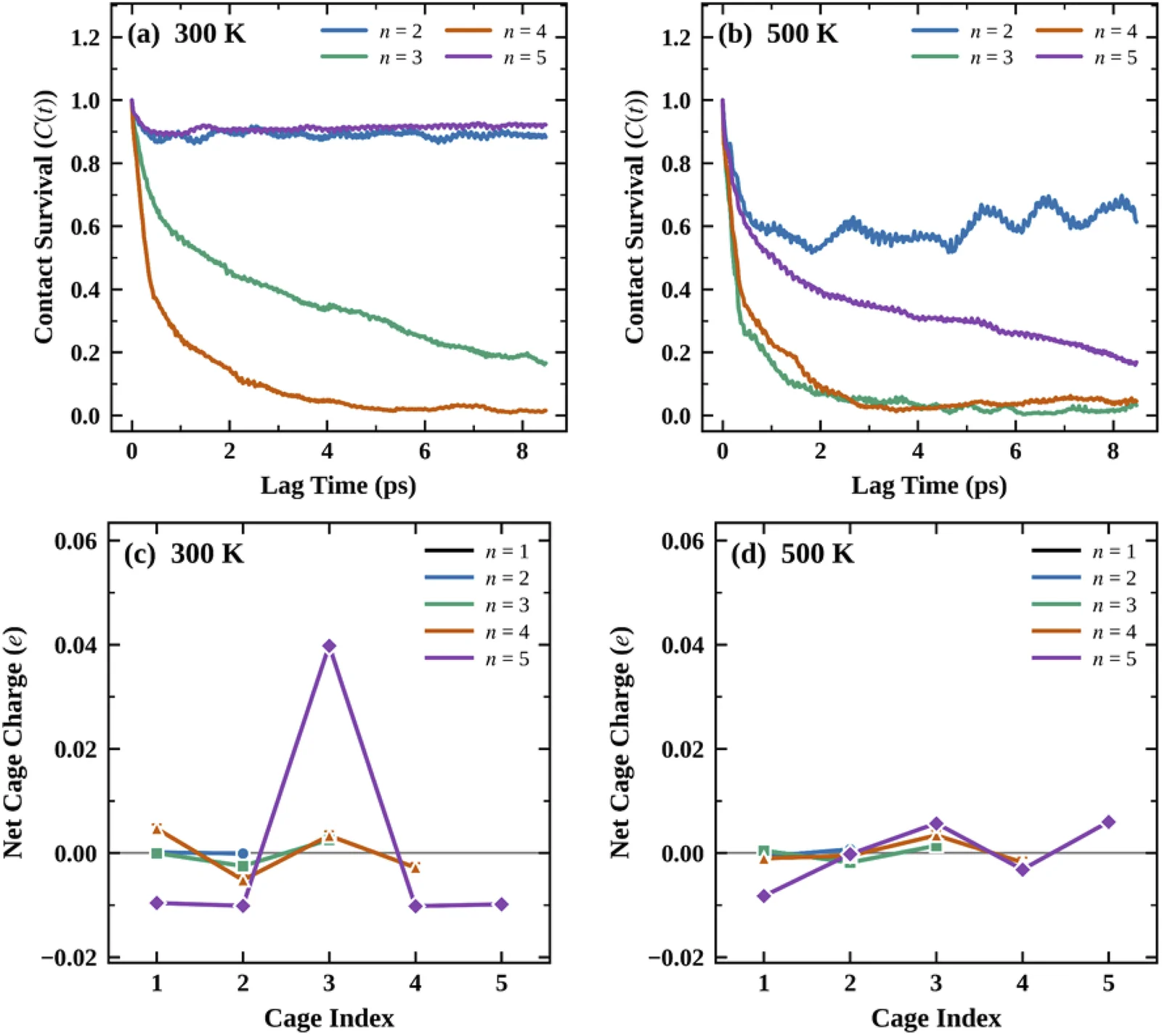
Inter-cage contact dynamics and charge distribution in (As_4_O_6_)_*n*_ clusters: contact autocorrelation functions *C*(*t*) at (a) 300 and (b)500 K, net charge of cage *vs.* cage index at (c) 300 and (d) 500 K, respectively.

### 3.6 Summary

This work examines whether the size-dependent evolution of C_*n*_ = (As_4_O_6_)_*n*_ clusters (*n* = 1–5) is a genuine cooperative stabilization or a simple additive size effect through five complementary structural, energetic, electronic, electrostatic and dynamical analyses. The basic structure of the individual As_4_O_6_ cages is retained throughout the series; the minimal changes in the As–O bond lengths (1.810–1.824 Å) and a very low distortion index indicates that aggregation takes place through non-covalent inter-cage interactions, but not through covalent re-structuring. Despite the rigidity of the structure, the energetic profile shows a non-linear behavior with a large increase in the binding energy per cage for C_5_ compared to the lower cluster sizes, suggesting that stability is determined by a cooperative packing efficiency and not by simple size scaling. This is an energetically active behavior that is consistently manifested in the electronic structure by a systematic shift in the character of the frontier orbitals with a corresponding trend in the global reactivity descriptors. These findings are further corroborated by charge and electrostatic analyses: Mulliken and Hirshfeld results combined with ΔMEP mapping indicate that electron redistribution is not confined to the fragments involved in the cluster assembly, but rather extends throughout the cluster, ultimately resulting in a pronounced charge accumulation in C_5_. Last but not least, MD simulations show that such a cooperative stabilization is not a static artefact since the C_5_ maintains a structural coherence and persistent inter-cage interactions even at higher temperatures. All these independent analyses lead to the consistent conclusion that the stability of (As_4_O_6_)_*n*_ clusters is dominated by non-additive cooperative interactions that arise as (As_4_O_6_)_*n*_ becomes larger and C_5_ is a symmetry-favored, cooperatively stabilized endpoint of the series.

## Author's contributions

Basma Ali: investigation, data curation, writing – original draft preparation. Abdul Majid: conceptualization, supervision, writing – review & editing. Muhammad Tayyab Raza: investigation, methodology, visualization, validation. Ali Hamza: investigation, data curation, methodology, software, resources, visualization. Waheed Almasry: methodology, resources, visualization, validation. Muhammad Usman: methodology, formal analysis, resources, visualization, validation. Sajjad Haider: methodology, resources, visualization, validation, funding acquisition.

## Conflicts of interest

There are no conflicts to declare.

## Supplementary Material

RA-OLF-D6RA08223H-s001

## Data Availability

The data supporting the findings of this article are given in the supplementary information (SI). Supplementary information: which contains the optimized geometries of all (As_4_O_6_)_*n*_ (*n* = 1–5) clusters, the SFO orbital-composition data, the reactivity descriptors, the multipole moments and the MD energy/temperature profiles. The computational input files, the MD trajectory files and the relevant data will be made available on request from the authors. See DOI: https://doi.org/10.1039/d6ra08223h.
