# Supplementary material for: Cooperative stabilization mechanism and electrostatic evolution in the zero dimensional arsenolite cluster aggregates: a first principles investigation
Source: RSC Adv. 2026 Oct 9;16(47):61988–2006. doi: 10.1039/d6ra08223h (PMC13651740; doi:10.1039/d6ra08223h)
Supplement: Supplementary file 1 [file RA-016-D6RA08223H-s001.pdf]

Supplementary Information (SI) for RSC Advances.
This journal is © The Royal Society of Chemistry

Cooperative Stabilization Mechanism and Electrostatic Evolution in the Zero Dimensional Arsenolite Cluster Aggregates: A First Principles Investigation

1.1 Structural Properties

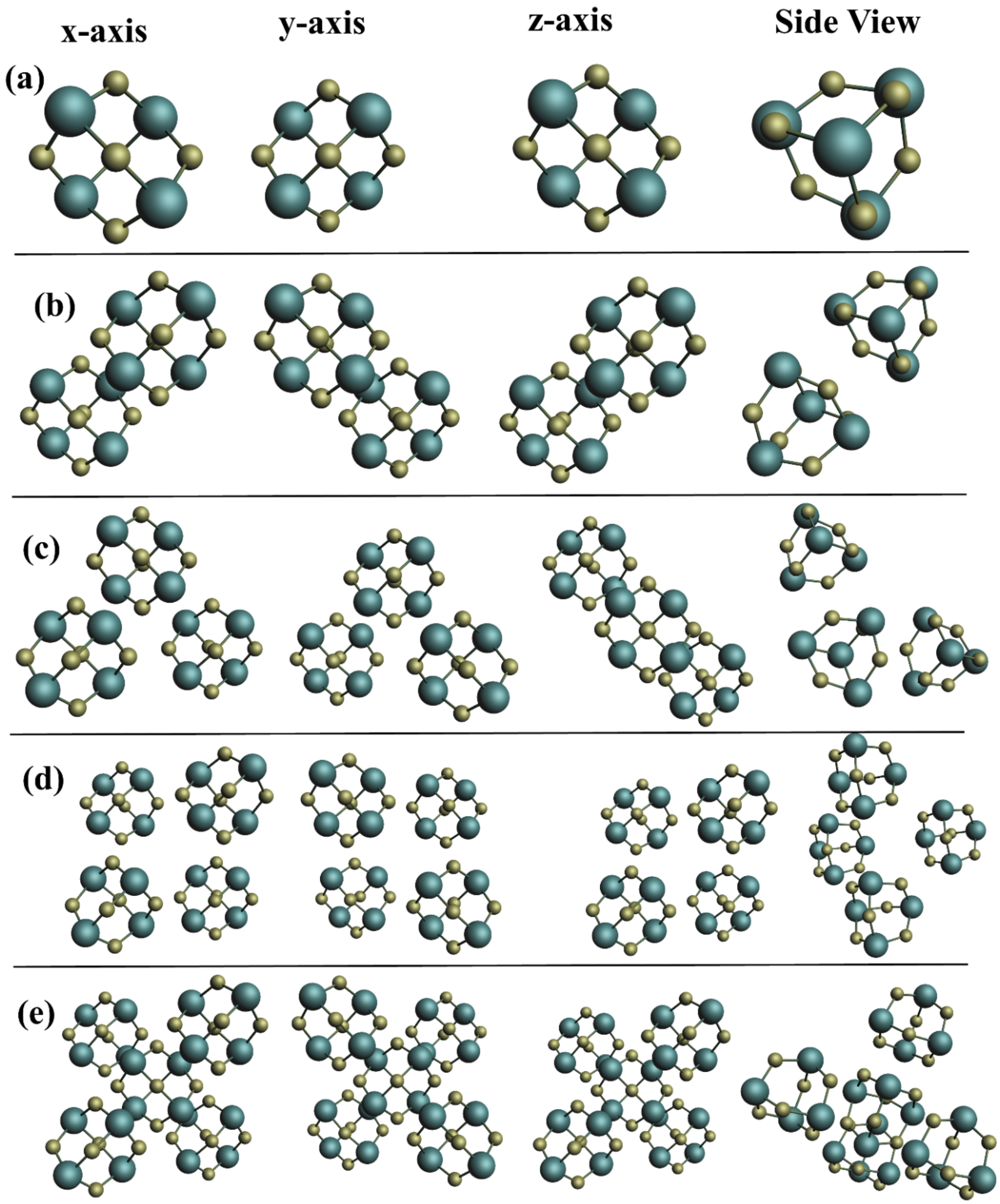

Figure S1: Three-dimensional structural representations of As_4O_6 cluster series (a)C1, (b)C2, (c)C3, (d)C4 and (e)C5 illustrating atomic arrangement and orientations also including side projections.

1.2 Quantitative Orbital Composition Descriptors

In order to make a systematic comparison of the frontier electronic structure during As_4O_6 cage aggregation, a series of quantitative descriptors was obtained from the symmetry fragment orbital (SFO) percentage contributions, which were derived from the ADF calculations [1]. The HOMO and LUMO compositions listed in Table S1, provide the percentage contributions of As and O atomic orbitals to the frontier molecular orbitals (FMO). Simultaneous comparison of several components of the orbitals across the series C₁–C₅ is more complicated, but these populations can be used to describe the electronic composition of each aggregate. Thus, the orbital populations were converted to a set of derived quantities that provide a physically interpretable summary of frontier orbital localization, orbital balance, oxygen participation, orbital purity and aggregation-induced orbital redistribution. The same types of chemically meaningful quantities derived from orbital analyses are used widely in studies of electronic structure and conceptual density functional theory [2, 3].

The LUMO Localization Index was defined as the total As contribution for the LUMO and can be given in the equation as,

$$\text{LUMO Localization Index} = C_{As}^{LUMO} \tag{1}$$

Where C_{As}^{LUMO} is the As total percentage contribution for the LUMO. This descriptor is a quantitative measure of localization of the frontier acceptor-orbital on arsenic atoms and allows an easy comparison of acceptor-state localization during cluster growth. The Relative s/p Orbital Balance was calculated as,

$$\text{Relative s/p Orbital Balance} = As_s^{HOMO} - As_p^{HOMO} \tag{2}$$

Where As_s^{HOMO} and As_p^{HOMO} represent the As-s and As-p contribution to the HOMO respectively. This descriptor represents the difference of s to p orbital participation of the arsenic atom and reflects the changes of the HOMO orbital hybridization during aggregation. The Oxygen p-Orbital Participation corresponds the O-p contribution to the HOMO and calculated as,

$$\text{Oxygen p - Orbital Participation} = O_p^{HOMO} \tag{3}$$

This descriptor directly assesses the participation of O lone pairs in the occupied frontier orbital and can be used to quantitatively follow the changes in participation of O lone pairs in aggregation. In order to reflect the extent of the frontier acceptor orbital that is made up of As-p orbitals, the LUMO p-Orbital Purity can be calculated as,

$$\text{LUMO p - Orbital Purity} = \frac{As_{p}^{LUMO}}{As_{Total}^{LUMO}} \times 100 \quad (4)$$

This descriptor indicates the degree of frontier orbital character that is maintained during cluster evolution.

Table S1: Elemental and orbital contribution (%) obtained from the SFO analysis of the FMO.

System	HOMO				LUMO				
	As		O		As			O	
	As-s	As-p	O-s	O-p	As-s	As-p	As-d	O-s	O-p
C_1	24.14	23.85	-	48.92	-	85.29	5.71	1.28	-
C_2	22.70	16.53	-	33.18	-	86.00	-	1.02	-
C_3	23.27	20.53	-	39.39	-	84.41	-	-	-
C_4	24.19	15.26	-	32.09	-	73.70	-	-	-
C_5	22.03	16.47	-	31.67	-	86.85	-	-	-

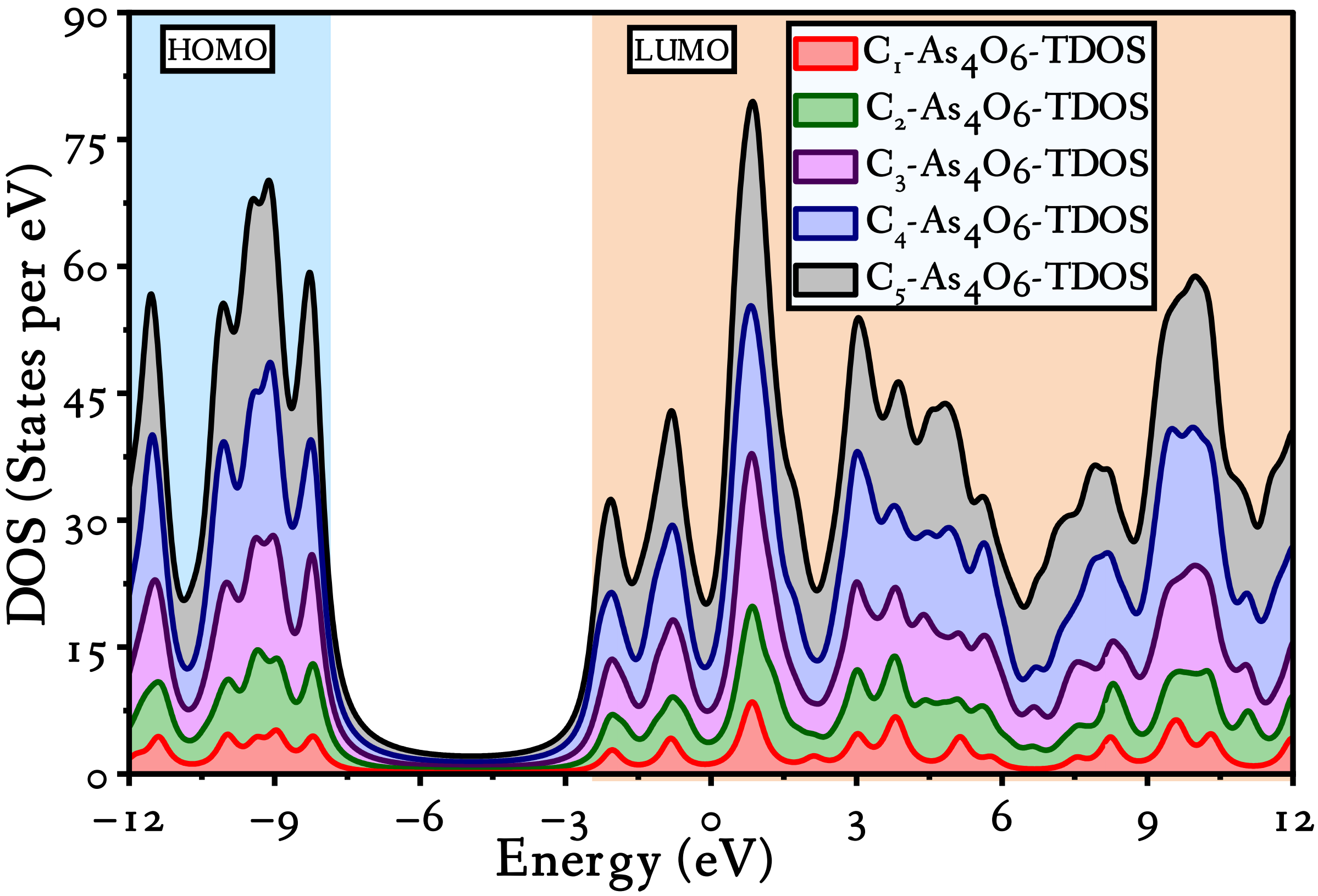

Figure S2: The evolution of electronic states with cluster size as shown for total DOS of (As₄O₆)ₙ clusters, C₁ to C₅.

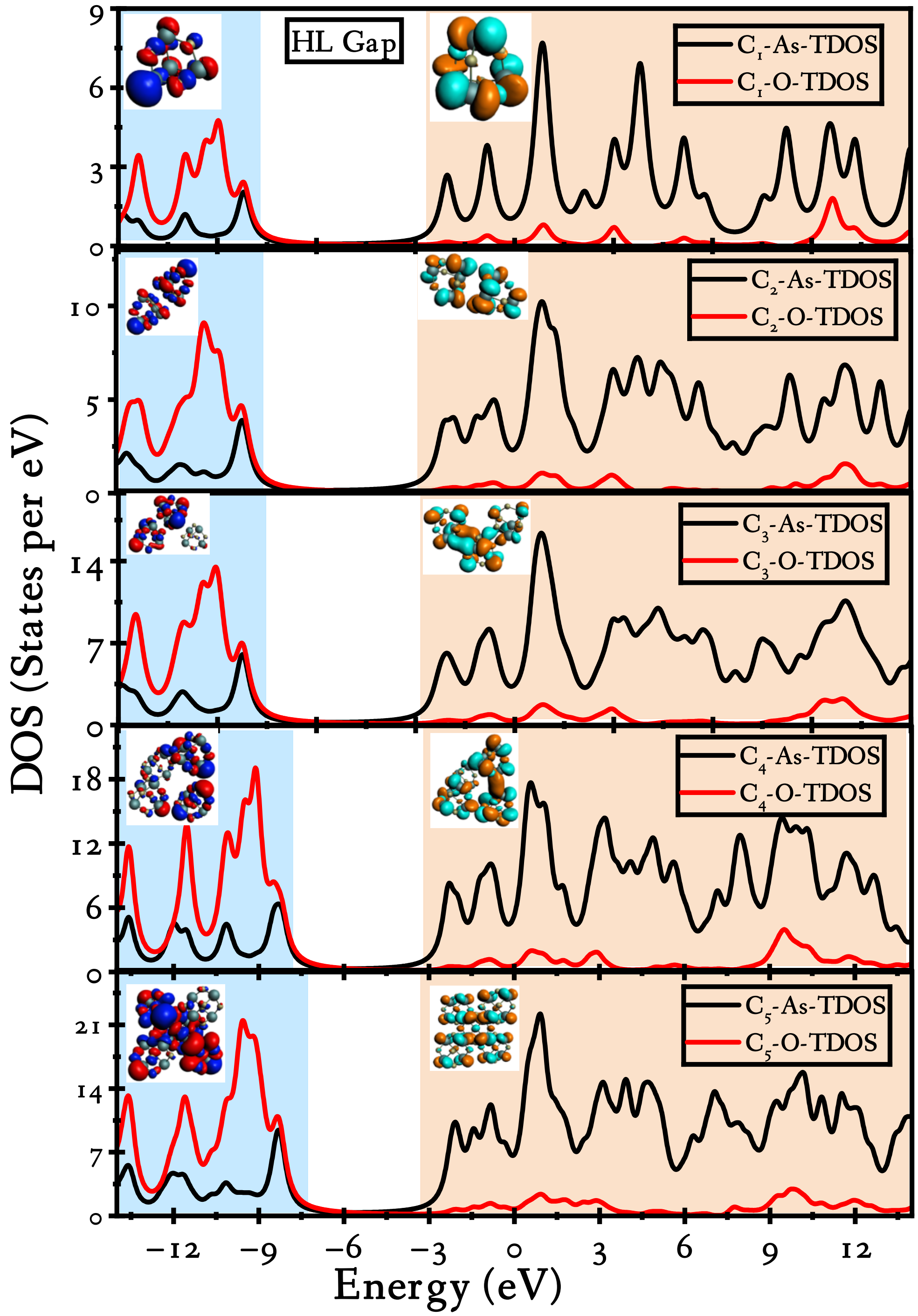

Figure S3: Energy-resolved features of electronic states of the C_n cluster as demonstrated by plots of total DOS peak distribution by As and O for HOMO and LUMO.

1.3 Reactivity Descriptors

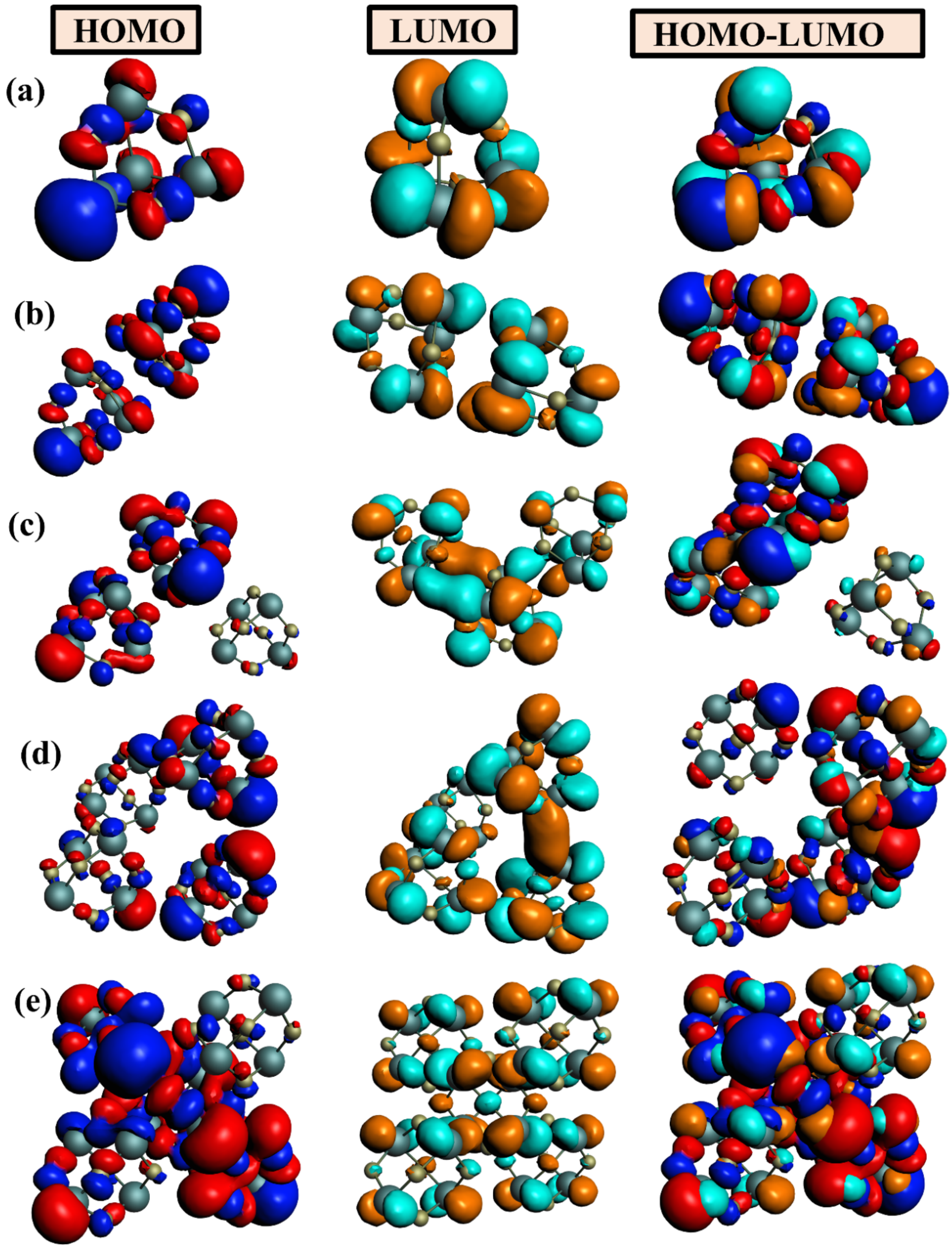

Figure S4: FMO isosurfaces Visualization of the As_4O_6 cluster evolution for (a)C_1, (b)C_2, (c)C_3, (d)C_4 and (e) C_5. Positive and negative phases of HOMO represented by the blue and red while LUMO is represented by the sky blue and brown color.

Table S2: Calculated orbital energies and reactivity descriptors of the As₄O₆ clusters.

Descriptors	C_1	C_2	C_3	C_4	C_5
HOMO (eV)	-8.199	-8.103	-8.173	-8.140	-8.178
LUMO (eV)	-2.041	-2.117	-2.314	-2.340	-2.246
HL gap (eV)	6.158	5.987	5.859	5.800	5.934
I (eV)	8.199	8.103	8.173	8.140	8.178
A (eV)	2.041	2.117	2.314	2.340	2.246
χ (eV)	5.120	5.110	5.244	5.240	5.213
μ (eV)	-5.120	-5.110	-5.244	-5.240	-5.213
η (eV)	3.079	2.993	2.930	2.900	2.967
S (eV^{-1})	0.162	0.167	0.171	0.172	0.169
ω (eV)	4.257	4.359	4.693	4.730	4.580
ΔN_{max}	1.663	1.707	1.790	1.807	1.757

1.4 Charge Analysis

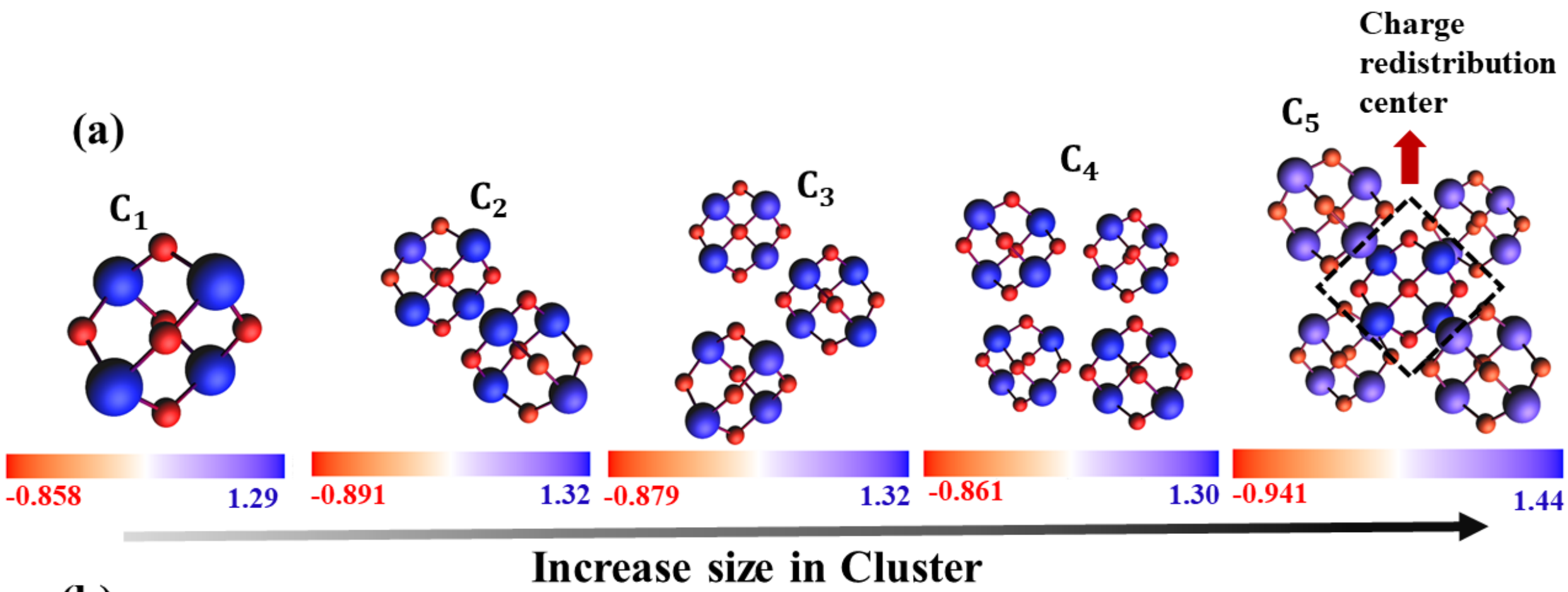

(b)

Parameter	As max (e)	As min (e)	O max (e)	O min (e)	Δq (e)	$\Delta q - \Delta q$_avg. $(C_1 - C_4)$ (e)	Incremental Δq (e)	%Δ
C_1	1.29	0.751	0.214	-0.858	2.148	-0.03175	-	-1.46
C_2	1.32	0.771	0.217	-0.891	2.211	+0.03125	+0.063	+1.43
C_3	1.32	0.770	0.220	-0.879	2.199	+0.01925	-0.012	+0.88
C_4	1.30	0.758	0.218	-0.861	2.161	-0.01875	-0.038	-0.86
C_5	1.44	0.847	0.251	-0.941	2.381	+0.20125	+0.220	+9.24

Figure S5: (a) Optimized geometries of (As₄O₆)ₙ clusters (C₁–C₅) with preserved cage framework and spatial charge distribution (b) mulliken population analysis summarizing variation of atomic charges and charge separation (Δq) with cluster sizes.

1.5 Molecular Multipole Moment

Table S3: Molecular dipole and quadrupole moment of the $C_n = (As_4O_6)_n$, n = 1 - 5 aggregation series.

System	Formula Unit	Dipole Moment (Debye)	Qxx (a.u.)	Qyy (a.u.)	Qzz (a.u.)
C_1	As_4O_6	0.002	0.000	0.000	0.000
C_2	As_8O_{12}	0.165	0.000	0.000	0.000
C_3	$As_{12}O_{18}$	0.235	-0.600	-0.506	1.105
C_4	$As_{16}O_{24}$	0.025	-0.148	0.144	0.003
C_5	$As_{20}O_{30}$	0.016	0.000	0.000	0.000

1.6 Thermal Stability

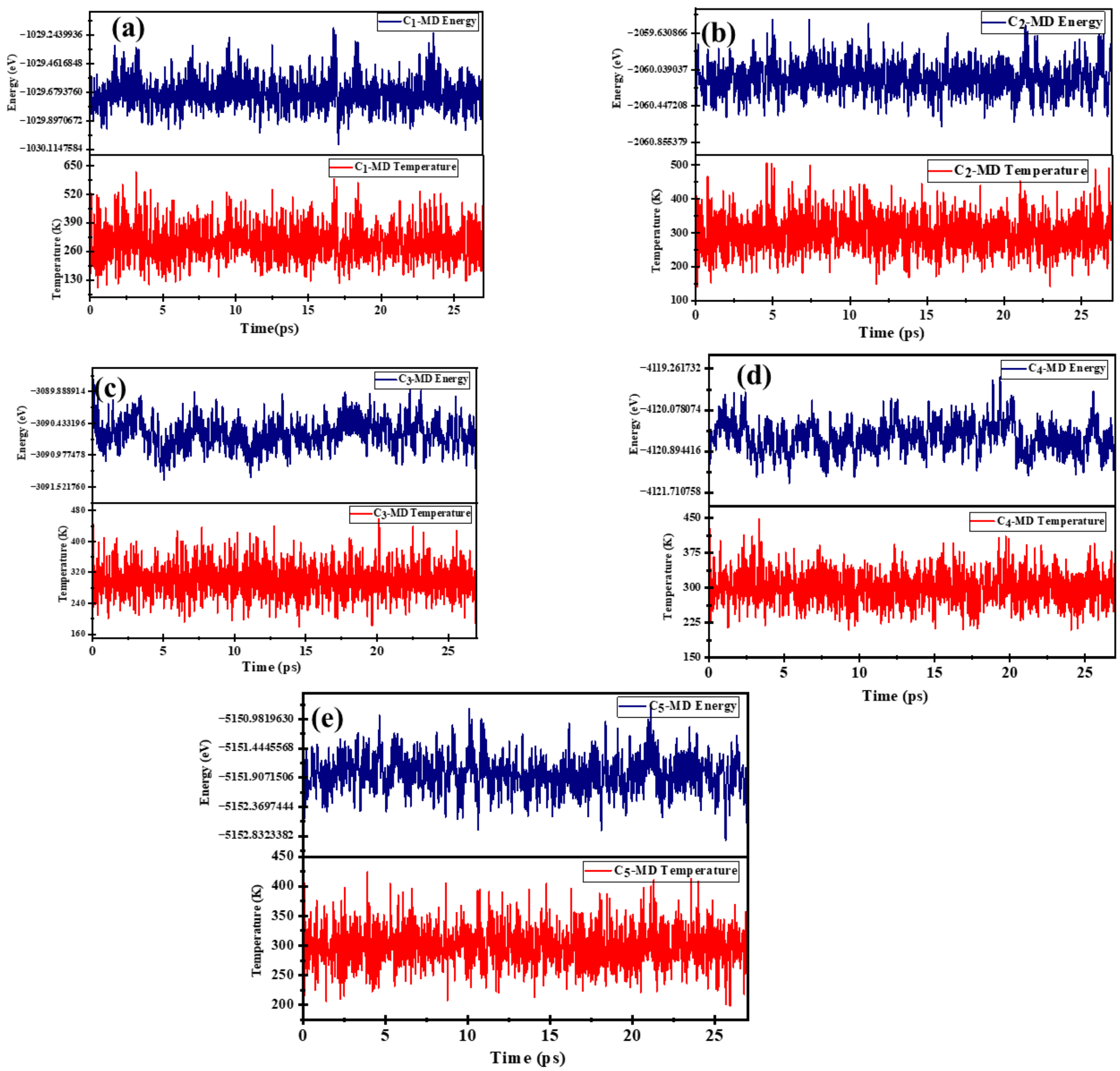

Figure S6: MD trajectories of the As_4O_6 clusters for (a)C_1, (b)C_2, (c)C_3, (d)C_4 and (e) C_5 at 300 K. Plot illustration of the energy variation with simulation time and also visualization of time profiles.

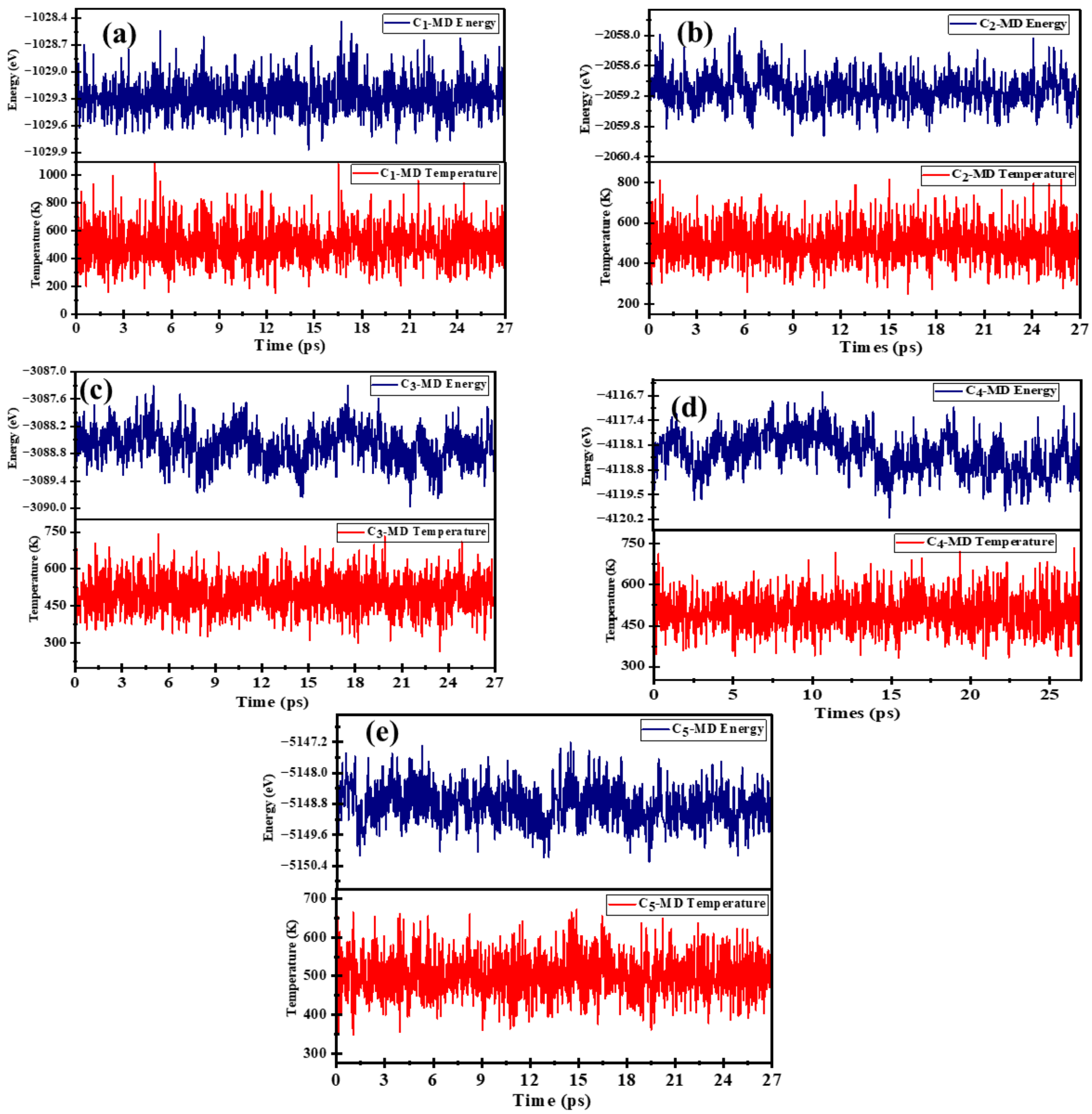

Figure S7: MD trajectories of the As_4O_6 clusters for (a)C_1, (b)C_2, (c)C_3, (d)C_4 and (e) C_5 at 500 K. Plot illustration of the energy variation with simulation time and also visualization of time profiles.

1.7 Trajectory Based Analysis

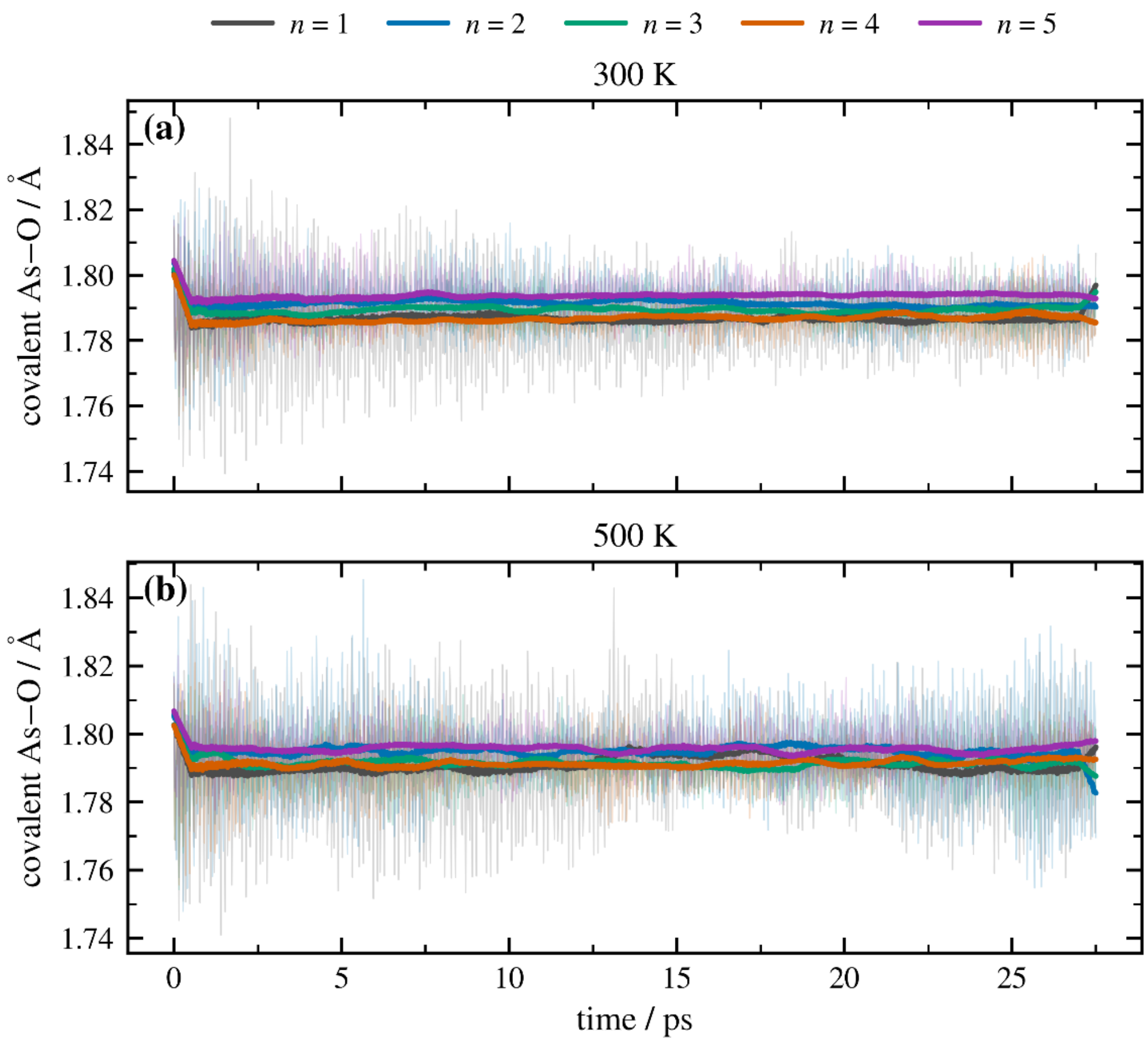

Figure S8: Time evolution of intra-cage As-O bond lengths in (As₄O₆)ₙ clusters at (a)300 K and (b)500 K.

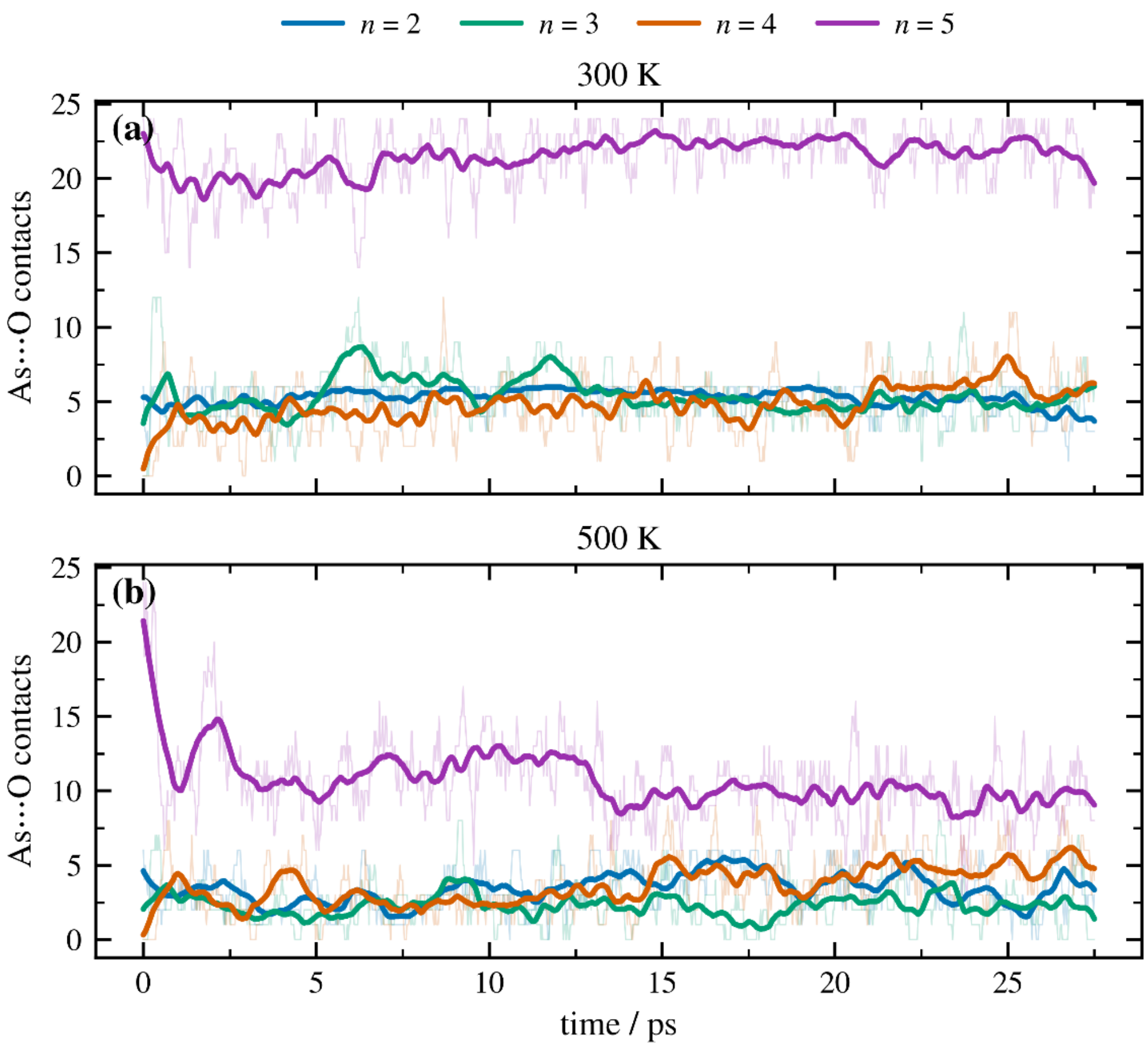

Figure S9: Time evolution of inter-cage As...O contact distances in (As₄O₆)ₙ clusters at (a) 300 K and (b) 500 K.

References

1. Wibowo-Teale, M., et al., Symmetry and reactivity of π-systems in electric and magnetic fields: a perspective from conceptual DFT. Physical Chemistry Chemical Physics, 2024. **26**(21): p. 15156–15180.
2. Meng, P., et al., Physics-informed transfer learning via frontier orbital pretraining for prediction of polymer electronic properties. The Journal of Chemical Physics, 2026. **164**(22).
3. Feng, Y., et al., Descriptors construction and application in catalytic site design. Iscience, 2025. **28**(8).
